# Both Rare and *De Novo* Copy Number Variants Are Prevalent in Agenesis of the Corpus Callosum but Not in Cerebellar Hypoplasia or Polymicrogyria

**DOI:** 10.1371/journal.pgen.1003823

**Published:** 2013-10-03

**Authors:** Samin A. Sajan, Liliana Fernandez, Sahar Esmaeeli Nieh, Eric Rider, Polina Bukshpun, Mari Wakahiro, Susan L. Christian, Jean-Baptiste Rivière, Christopher T. Sullivan, Jyotsna Sudi, Michael J. Herriges, Alexander R. Paciorkowski, A. James Barkovich, Joseph T. Glessner, Kathleen J. Millen, Hakon Hakonarson, William B. Dobyns, Elliott H. Sherr

**Affiliations:** 1 Department of Pediatrics, Section of Neurology, Baylor College of Medicine, Houston, Texas, United States of America; 2 Department of Neurology, University of California, San Francisco, San Francisco, California, United States of America; 3 Center for Integrative Brain Research, Seattle Children's Research Institute, Seattle, Washington, United States of America; 4 Equipe Génétique des Anomalies du Dévelopement, Université de Bourgogne, Dijon, France; 5 Department of Human Genetics, University of Chicago, Chicago, Illinois, United States of America; 6 Department of Cell and Developmental Biology, University of Pennsylvania, Philadelphia, Pennsylvania, United States of America; 7 Departments of Neurology, Pediatrics, and Biomedical Genetics, University of Rochester Medical Center, Rochester, New York, United States of America; 8 Department of Radiology and Biomedical Imaging, Division of Neuroradiology, University of California, San Francisco, San Francisco, California, United States of America; 9 Center for Applied Genomics, Children's Hospital of Philadelphia, Philadelphia, Pennsylvania, United States of America; 10 Department of Pediatrics, University of Washington, Seattle, Washington, United States of America; 11 Department of Pediatrics, the Perelman School of Medicine, University of Pennsylvania, Philadelphia, Pennsylvania, United States of America; 12 Department of Neurology, University of Washington, Seattle, Washington, United States of America; Massachusetts General Hospital, United States of America

## Abstract

Agenesis of the corpus callosum (ACC), cerebellar hypoplasia (CBLH), and polymicrogyria (PMG) are severe congenital brain malformations with largely undiscovered causes. We conducted a large-scale chromosomal copy number variation (CNV) discovery effort in 255 ACC, 220 CBLH, and 147 PMG patients, and 2,349 controls. Compared to controls, significantly more ACC, but unexpectedly not CBLH or PMG patients, had rare genic CNVs over one megabase (p = 1.48×10^−3^; odds ratio [OR] = 3.19; 95% confidence interval [CI] = 1.89–5.39). Rare genic CNVs were those that impacted at least one gene in less than 1% of the combined population of patients and controls. Compared to controls, significantly more ACC but not CBLH or PMG patients had rare CNVs impacting over 20 genes (p = 0.01; OR = 2.95; 95% CI = 1.69–5.18). Independent qPCR confirmation showed that 9.4% of ACC patients had *de novo* CNVs. These, in comparison to inherited CNVs, preferentially overlapped *de novo* CNVs previously observed in patients with autism spectrum disorders (p = 3.06×10^−4^; OR = 7.55; 95% CI = 2.40–23.72). Interestingly, numerous reports have shown a reduced corpus callosum area in autistic patients, and diminished social and executive function in many ACC patients. We also confirmed and refined previously known CNVs, including significantly narrowing the 8p23.1-p11.1 duplication present in 2% of our current ACC cohort. We found six novel CNVs, each in a single patient, that are likely deleterious: deletions of 1p31.3-p31.1, 1q31.2-q31.3, 5q23.1, and 15q11.2-q13.1; and duplications of 2q11.2-q13 and 11p14.3-p14.2. One ACC patient with microcephaly had a paternally inherited deletion of 16p13.11 that included *NDE1*. Exome sequencing identified a recessive maternally inherited nonsense mutation in the non-deleted allele of *NDE1*, revealing the complexity of ACC genetics. This is the first systematic study of CNVs in congenital brain malformations, and shows a much higher prevalence of large gene-rich CNVs in ACC than in CBLH and PMG.

## Author Summary

Here, we systematically test the genetic etiology of three common developmental brain malformations: agenesis of the corpus callosum (ACC), cerebellar hypoplasia (CBLH), and polymicrogyria (PMG) by copy number variation (CNV) analysis in a large cohort of brain malformation patients and controls. We found significantly more ACC but not CBLH or PMG patients with rare genic CNVs over one megabase and with rare CNVs impacting over 20 genes when compared with controls. *De novo* CNVs were found in 9.4% of ACC patients, and interestingly many such CNVs overlapped with *de novo* CNVs observed in autism. Notably, numerous studies have demonstrated a reduction in the corpus callosum area in autistic brains. Our analysis also refined previously known large CNVs that cause these malformations, and identified six novel CNVs that are likely deleterious. One ACC patient had inherited a deletion from the father which, through exome sequencing, was found to uncover a recessive nonsense mutation in *NDE1* on the non-deleted allele inherited from the mother. Our study is the first to systematically evaluate the burden of rare genic CNVs in congenital brain malformations and shows that large gene-rich CNVs are more common in ACC than in CBLH and PMG.

## Introduction

Agenesis of the corpus callosum (ACC), cerebellar hypoplasia (CBLH), and polymicrogyria (PMG) are a group of complex, severe, and causally heterogeneous brain malformations that result in significant developmental disability and seizures, and sometimes occur together in the same individual. Even individuals with ACC who have intelligent quotients (IQs) in the normal range often have deficits in social and executive functioning and may have an autism spectrum disorder (hereafter autism) [1], [2]. While the incidence of each malformation is low (∼1/4000 live births, with PMG even less prevalent) [3]–[5], they are nevertheless the most common developmental brain malformations encountered in the clinic and both ACC and CBLH are frequently seen in prenatal brain imaging [6], [7].

Numerous clinical reports or studies focused on individual loci have shown that genomic copy number variants – particularly those that are several megabases in length, affect many genes, and arise *de novo* – are implicated in the etiology of these three brain malformations. For instance, recurrent CNVs in 1p36, 1q42-43 and 8p23, have been identified in a number of ACC patients, and we recently reviewed more than 40 others mostly detected by karyotype analysis [3], [8]–[14]. Some CNVs resulting in CBLH have been found in 3q22.3-q25.2, 6p25.3, 13q12.3-q14.11, 22q13 and Xq28, with the causative genes in 6p25.3 (*FOXC1*) and 3q22.3-q25.2 (*ZIC1* and *ZIC4*) having been identified [15]–[20]. CNVs in PMG have been mapped to 1p36.3, 2p16.1-p23.1, 4q21.21-q22.1, 6q26-q27, and 22q11.2, among others [4], [21]–[23]. Most of these studies have used isolated patients or small series, although one study examined rare CNVs in structural brain malformations in 169 patients. However, that study did not distinguish among patients with different classes of malformations (focal cortical dysplasia, microcephaly, lissencephaly, posterior fossa defects, and callosal agenesis, with the latter found in only 18 patients) and focused mostly on pathway analysis [13]. Thus, no studies have assessed the genome-wide burden of rare CNVs or the rate of *de novo* CNVs in a systematic manner in patients with brain malformations.

Overcoming the limitations of prior studies, we analyzed genic CNVs in 545 patients with one or more of these three common brain malformations and in 2,349 control individuals. We hypothesized that these patients have a higher genome-wide burden of large rare genic CNVs than controls and that a significant percentage of them have large *de novo* likely pathogenic CNVs including both novel and previously reported intervals. We further anticipated that many of the *de novo* CNVs would be sub-microscopic [less than 4 megabases (Mb)], demonstrating a larger disease burden for CNVs than previously seen through cytogenetic approaches.

## Results

### CNV Identification and Analysis

**Figure 1:**
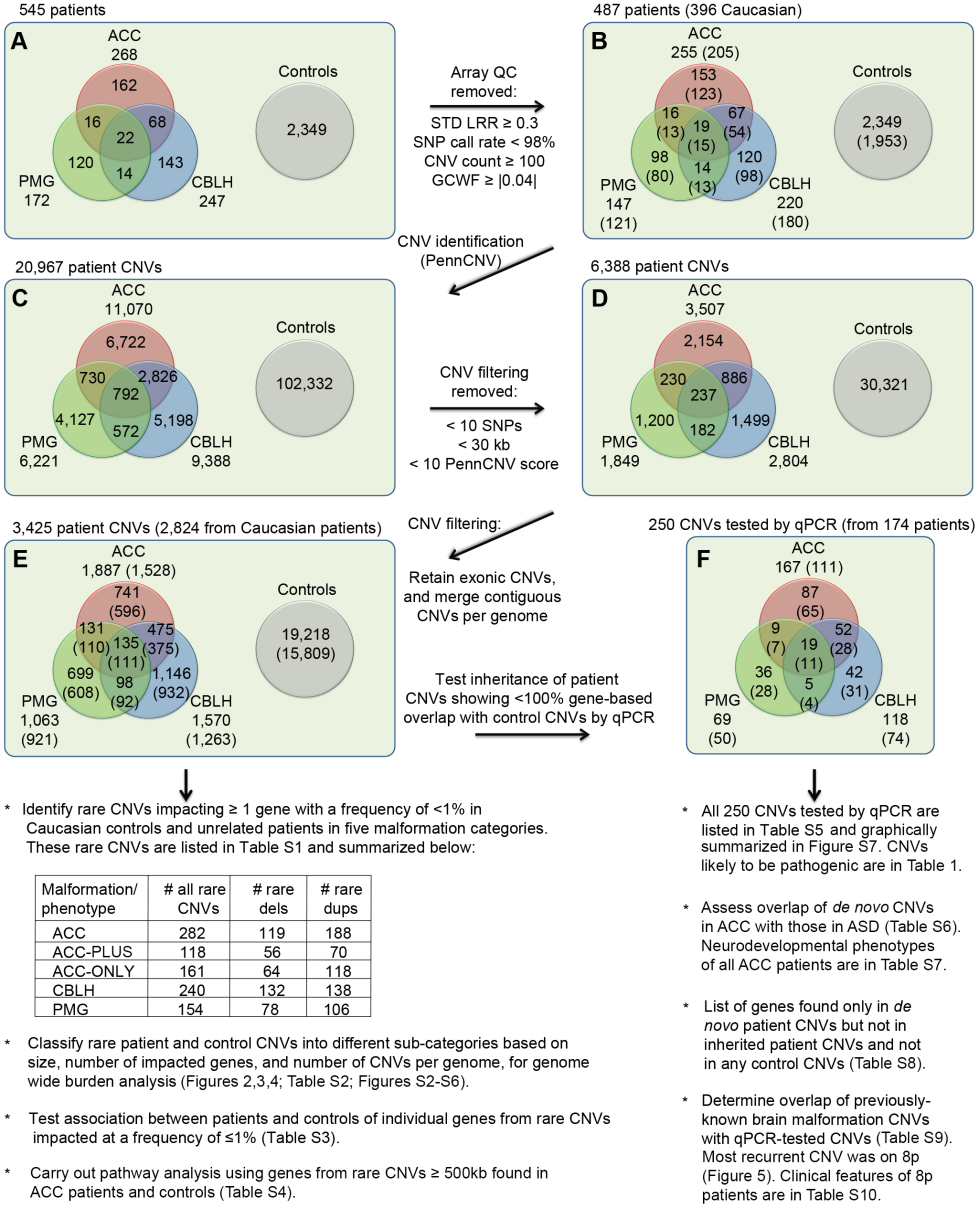
Outline of CNVs analysis. A total of 545 patients with at least one of three brain malformations (agenesis of the corpus callosum or ACC, cerebellar malformations or CBLH, and polymicrogyria or PMG) and 2,349 controls were genotyped for CNVs identification. Panel A shows the initial numbers of patients with different malformations whereas panel B shows numbers that remained following the application of array quality control (QC) criteria. Numbers in parentheses represent patients of Caucasian ethnicity. Panels C, D, and E show the numbers of CNVs in patients and controls that remained at different stages of filtering. The numbers in parentheses in panel E denote CNVs from Caucasian patients/controls that were used to identify rare CNVs and carry out subsequent analyses shown underneath, whereas CNVs from all patients (Caucasian and non-Caucasian) were compared with control CNVs to identify patient-specific CNVs. Confirmation and determination of inheritance (where parents were available) for this latter list of CNVs were done by quantitative PCR (qPCR). Panel F shows the numbers of these CNVs in each malformation, with the numbers of patients shown in parentheses. Additional analyses, shown below panel F, were also carried out using these qPCR-validated CNVs. Abbreviations: STD LRR, standard deviation of the logarithm of the ratio of fluorescence of probes of the two alleles of a given SNP on the array; GCWF, GC wave factor.

We genotyped 545 patients diagnosed with ACC, CBLH or PMG on the Illumina InfiniumII HumanHap610 SNP array. Because these brain malformations often co-occur, our cohort included 120 patients diagnosed with at least two of these three malformations (Figure 1). Throughout the rest of the text we use ACC to refer to all patients who have agenesis of the corpus callosum regardless of whether they also have CBLH, PMG or both. We further divided the ACC cohort into two groups to distinguish those who also had CBLH or PMG (called ACC-PLUS) from those who did not (called ACC-ONLY). The terms CBLH and PMG refer to patients diagnosed with CBLH or PMG, respectively, regardless of any other malformation. Consequently, a patient diagnosed with both CBLH and PMG was analyzed twice – once with the CBLH group of patients and again with the PMG group of patients. We used control data from 2,349 neurologically normal individuals who had been recruited and genotyped previously on the same platform at Children's Hospital of Philadelphia. After applying array quality control (QC) measures, 487 of 545 patients (of whom 396 were Caucasian) were considered suitable for CNV identification and downstream analyses (Figure 1). The 2,349 control arrays, including 1,953 from Caucasian individuals, passed identical QC measures.

Using PennCNV [24], we found 2,879 rare genic CNVs in controls of which 73 (2.5%) were over 1 Mb. Rare CNVs were those that impacted at least one exon of a gene at a frequency of less than 1% in the combined population of patients and controls. Compared to these control CNVs, ACC patients had 282 rare CNVs of which significantly more were over 1 Mb (25 CNVs or 8.9%, p = 5.19×10^−7^; odds ratio [OR] = 3.74; 95% confidence interval [CI] = 2.33–5.99). However, rare CNVs over 1 Mb were not enriched in CBLH and PMG patients (9 out of 240 CNVs or 3.8%, p = 0.19 and 6 out of 154 CNVs or 3.9%, p = 0.19, respectively). All rare CNVs in patients are in Table S1.

We tested whether this observed lack of significantly more large rare CNVs in CBLH and PMG could be explained by the smaller numbers of these patients compared to ACC patients. We used 205 ACC, 180 CBLH, and 121 PMG patients of Caucasian ethnicity, and 1,953 controls (also Caucasian) to determine the overall burden of rare genic CNVs. Of these, 20 (9.8%), 7 (3.9%), 6 (5.0%), and 64 (3.3%), respectively, had at least one rare CNV over 1 Mb, indicating that significantly more ACC (p = 4.49×10^−5^) but not CBLH (p = 0.42) and PMG (p = 0.19) patients compared to controls had large rare CNVs. Our power analysis (see Materials and Methods) showed that the achieved power, which can be thought of as the probability of detecting a true positive result, for observing this significant difference between ACC and controls was 0.96. If CBLH and PMG patients truly had the same level of enrichment of rare CNVs over 1 Mb as ACC patients then we would have detected it using our current patient population with confidence levels of 0.95 and 0.87, respectively (Figure S1), strongly suggesting that this lower rate of large CNVs in CBLH and PMG cannot be fully explained by our patient numbers.

### Genome-wide Burden of Rare Genic CNVs

**Figure 2:**
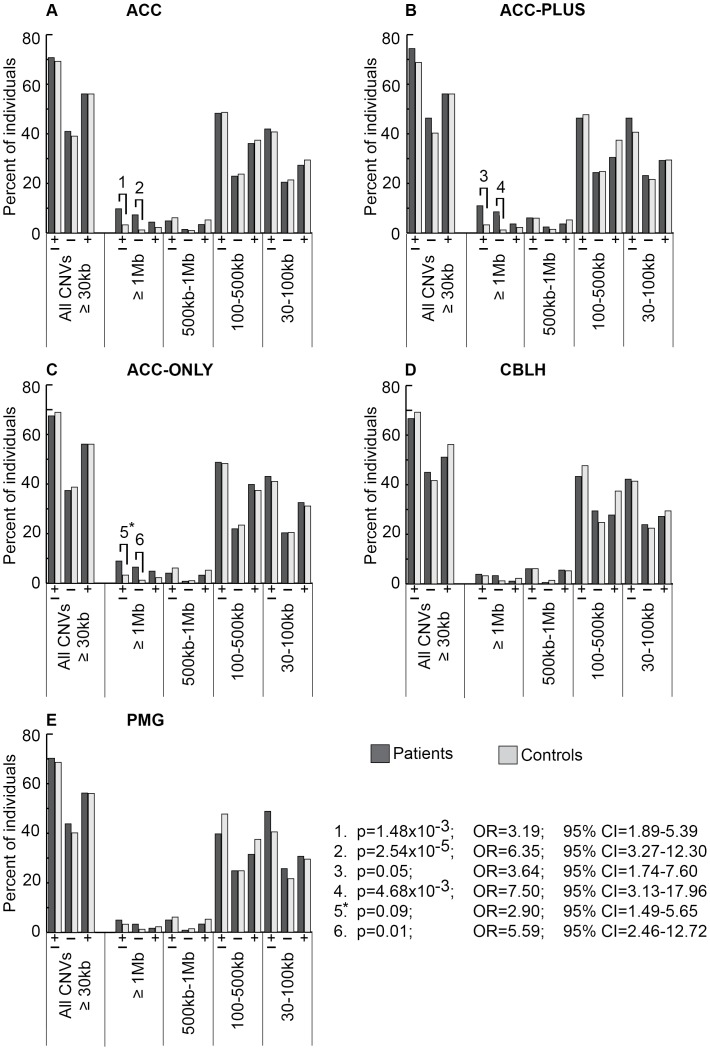
Genome-wide burden of rare CNVs based on size in brain malformation patients and controls of Caucasian ethnicity. There were 205 ACC (panel A), 82 ACC-PLUS (panel B), 123 ACC-ONLY (panel C), 180 CBLH (panel D), and 121 PMG (panel E) patients, and 1,953 controls. In each malformation we examined all rare CNVs that were at least 30 kb (all CNVs≥30 kb), followed by those that were at least 1 Mb (≥1 Mb), those that were at least 500 kb but less than 1 Mb (500 kb–1 Mb), those that were at least 100 kb but less than 500 kb (100–500 kb), and those that were at least 30 kb but less than 100 kb (30–100 kb). Deletions are represented by “−” and duplications by “+”. Significant differences between patients (dark bars) and controls (light bars) are shown by black lines/hooks that connect patients and controls with numbers listed above. The numbers correspond to corrected p-values, odds ratios (OR), and 95% confidence intervals (CI) provided in the lower right. Asterisk: while the corrected p-value was not significant (0.09), the odds ratio (2.90) and 95% confident interval (1.49–5.65) were both highly suggestive of a significant difference.

**Figure 3:**
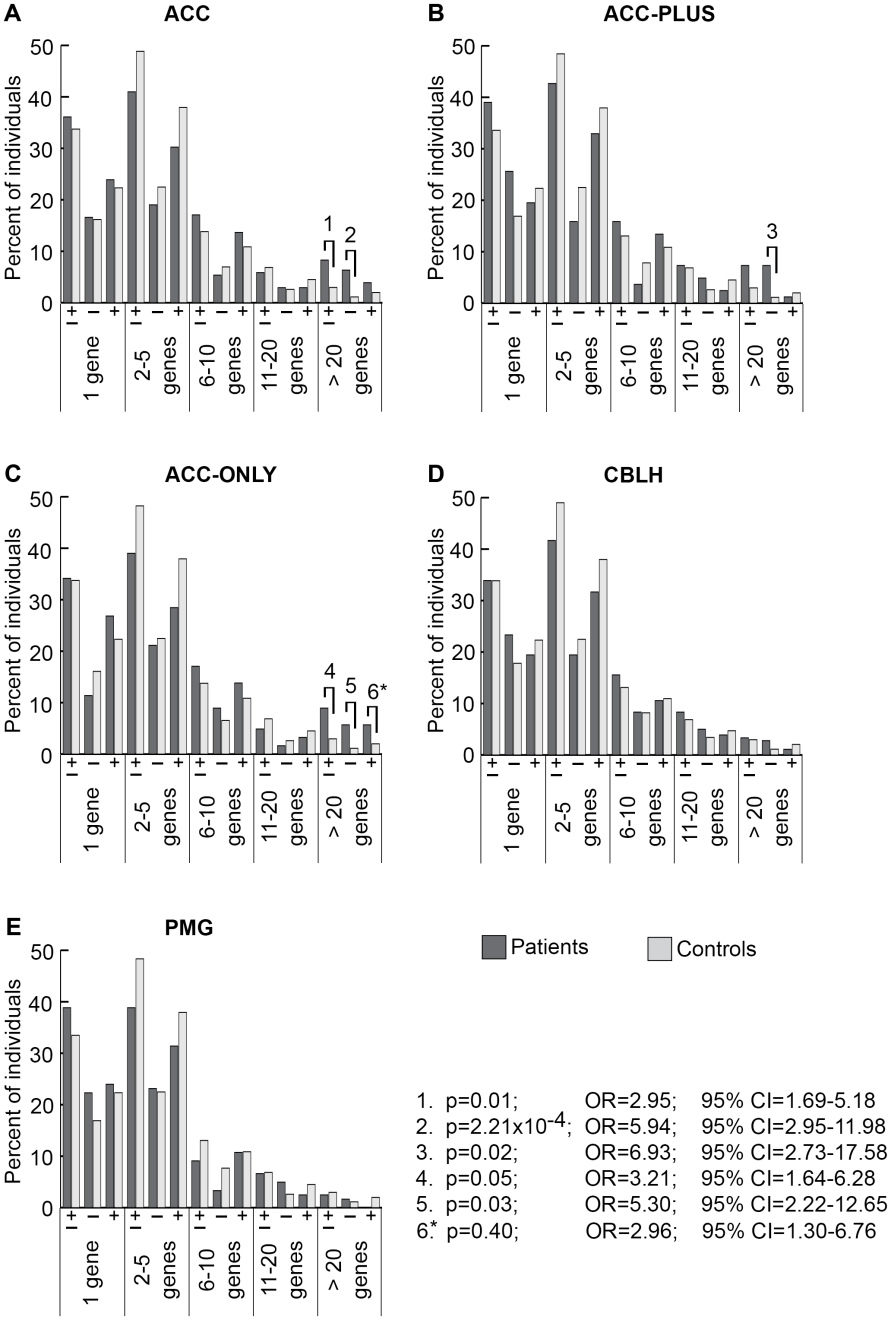
Genome-wide burden of rare CNVs based on number of genes impacted in brain malformation patients and controls of Caucasian ethnicity. There were 205 ACC (panel A), 82 ACC-PLUS (panel B), 123 ACC-ONLY (panel C), 180 CBLH (panel D), and 121 PMG (panel E) patients, and 1,953 controls. Deletions and duplications were assessed together (“±”) and also separately (“−” and “+”, respectively). Significant differences between patients (dark bars) and controls (light bars) are shown by black lines/hooks that connect patients and controls with numbers listed above. The numbers correspond to corrected p-values, odds ratios (OR), and 95% confidence intervals (CI) provided in the lower right. Asterisk: while the corrected p-value was not significant (0.40), the odds ratio (2.96) and 95% confident interval (1.30–6.76) were both highly suggestive of a significant difference.

We determined the genome-wide burden of rare genic CNVs in patients and controls based on three criteria: CNV size (Figure 2), number of genes with at least one exon impacted (Figure 3), and number of CNVs per genome (Figures S2, S3, S4, S5, S6 and Table S2). We found a significant increase in rare CNV burden using the first two criteria, but not the third, in ACC patients relative to controls. The rare CNV burden in CBLH and PMG patients was not significantly different from controls using any of these three criteria.

Based on CNV size, a significantly higher proportion of ACC patients had at least one CNV≥1 Mb (Figure 2A, p-value #1: p = 1.48×10^−3^; OR = 3.19; 95% CI = 1.89–5.39). This was particularly true for deletions (Figure 2A, p-value #2: p = 2.54×10^−5^; OR = 6.35; 95% CI = 3.27–12.30), but not duplications. Smaller size classes of CNVs (less than 1 Mb), regardless of whether deletions and duplications were analyzed separately or together, did not show a significant difference. We then split ACC patients into two subgroups to determine whether this observation was due to the presence of ACC together with CBLH and/or PMG (ACC-PLUS, Figure 2B) or due to isolated ACC (ACC-ONLY, Figure 2C). Both subgroups exhibited similar trends of rare CNV burden based on size, suggesting that callosal agenesis was the defining feature. CBLH (Figure 2D) and PMG (Figure 2E) patients did not have a significantly higher burden of rare CNVs from any size class.

Based on gene number, significantly more ACC patients had rare CNVs impacting over 20 genes (Figure 3A, p-value #1: p = 0.01; OR = 2.95; 95% CI = 1.69–5.18). This was also the case for deletions analyzed alone (Figure 3A, p-value #2: p = 2.21×10^−4^; OR = 5.94; 95% CI = 2.95–11.98), but not for duplications alone, suggesting that deletions that impact many genes may be more pathogenic. Both ACC-PLUS and ACC-ONLY subgroups displayed similar trends (Figure 3B and 3C, respectively), except that duplications impacting over 20 genes in ACC-PLUS were not significantly different than controls. And even though duplications were not significant after correcting for multiple tests in ACC-ONLY as well, it is highly likely that they are enriched in these patients based on the odds ratio (Figure 3C, p-value #6: p = 0.40; OR = 2.96; 95% CI = 1.30–6.76). This is in agreement with deletions being more deleterious since ACC-PLUS is often a more severe phenotype than ACC-ONLY. Patients with CBLH (Figure 3D) and PMG (Figure 3E) did not have a significantly higher burden of rare CNVs based on any gene number.

Based on number of CNVs per genome, we did not find any significant difference in the rare CNV burden between patients and controls (Figures S2, S3, S4, S5, S6).

**Figure 4:**
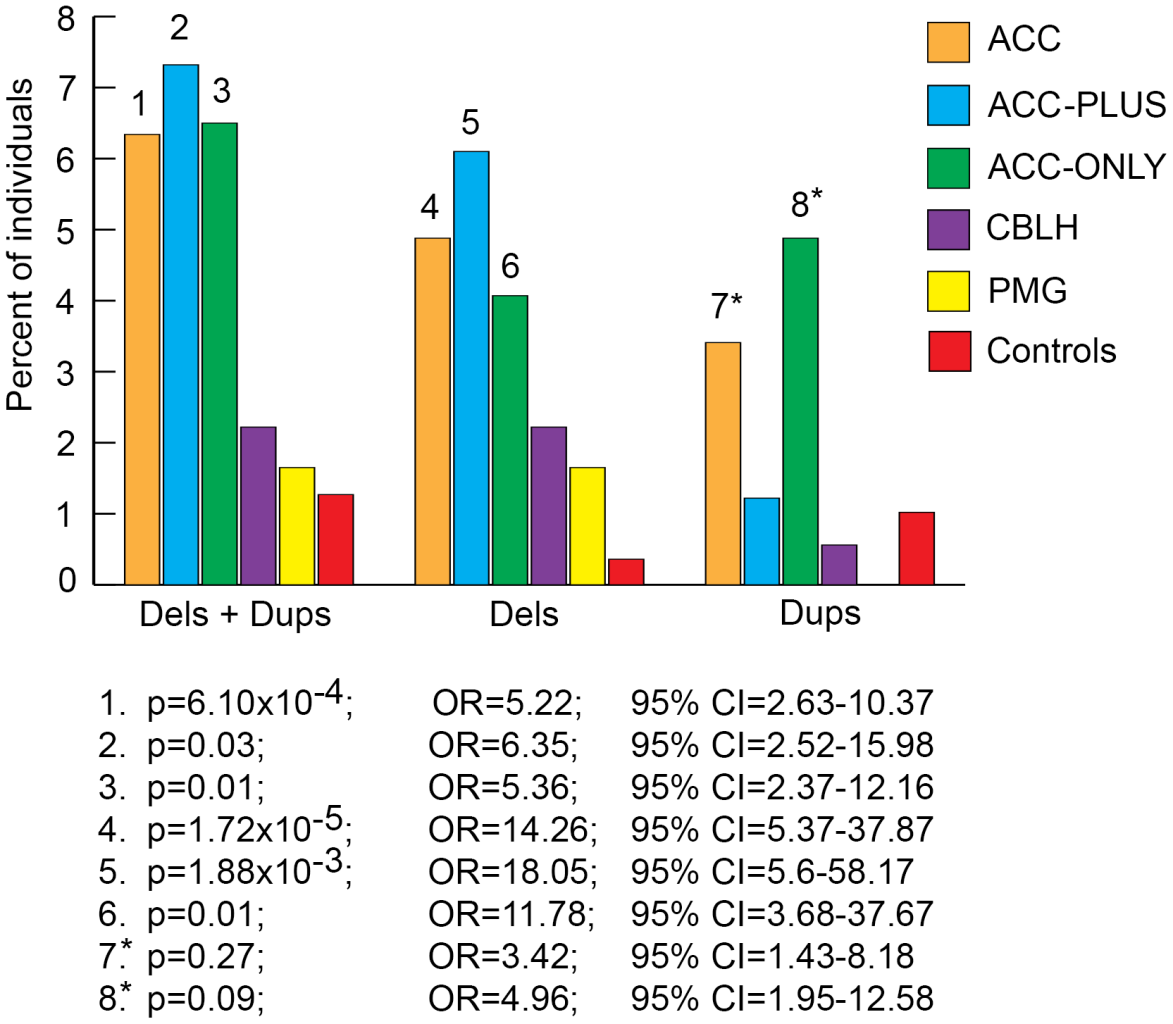
Genome-wide burden of rare CNVs≥1 Mb that impacted ≥20 genes in brain malformation patients and controls. Deletions and duplications were assessed together (“±”) and also separately (“−” and “+”, respectively). Significant differences are shown by numbers on top of bars for the respective malformations. These numbers correspond to corrected p-values, odds ratios (OR), and 95% confidence intervals (CI) provided below the graph. Asterisks: while the corrected p-values were not significant (>0.05), the odds ratios and 95% confident intervals were both highly suggestive of significant differences between patients and controls.

We next split each size class of rare CNVs into sub-classes based on number of genes impacted and number of CNVs per genome and determined the burden of each sub-class in patients and controls. The most significant result from this analysis was that rare CNVs over 1 Mb impacting more than 20 genes were highly enriched in ACC, ACC-PLUS, and ACC-ONLY patients (Figure 4 and Figures S2, S3, S4). As before, duplications by themselves were not enriched in ACC-PLUS. The burden of these various sub-classes of CNVs was not significantly different in CBLH and PMG patients when compared with controls (Figures S5 and S6).

### Gene Content of Rare CNVs

**Figure 5:**
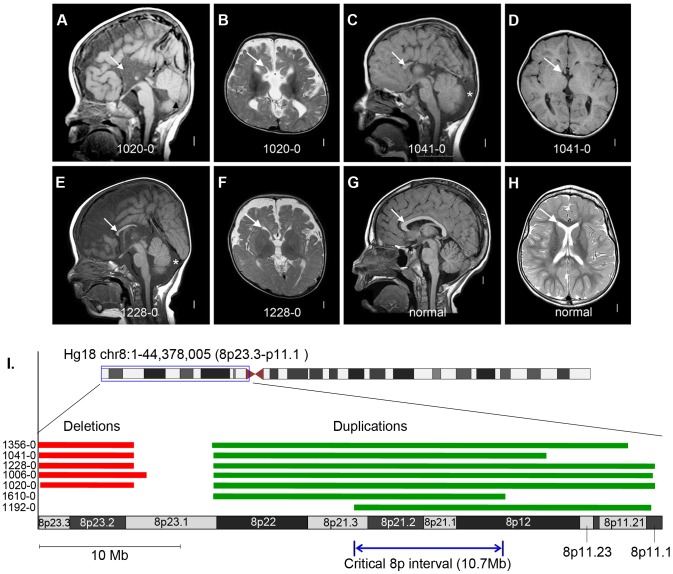
Brain magnetic resonance imaging and critical region analysis of the duplication-deletion 8p syndrome. Panels A–F show brain images of three select patients with ACC who had large *de novo* CNVs on chromosome 8p, and panels G–H show brain images of a normal individual. Midline sagittal images (panels A, C, E) demonstrate complete (panel A) or partial (panels C and E) agenesis of the corpus callosum indicated by white arrows. In panel A the black arrowhead points to mild hypoplasia and upward rotation of the cerebellar vermis, and in panels C and E the asterisks show enlarged cisterna magna below and behind the cerebellum. Axial images of patients at the level of the thalamus demonstrate enlarged extra-axial fluid over the frontal lobes and mildly enlarged 3rd ventricles (panels B and F). In the patient with complete agenesis, the lateral ventricles are also mildly enlarged and widely separated (panel B). Panel I shows the genomic locations of the chromosome 8p CNVs in seven patients with ACC, with patients 1610-0 and 1192-0 being instrumental in defining the 10.7 Mb critical region (Hg18 chr8:22609566-33311183). Table S10 lists the clinical features of all seven patients.

We next asked whether individual genes within rare CNVs were deleted or duplicated more often in patients than controls. Genes impacted in controls but never in patients were not included in this analysis. ACC and CBLH, but not PMG patients, had one or more genes that survived multiple testing correction (Benjamini-Hochberg false discovery rate of ≤0.05). Table S3 lists a subset of these significant genes that were either deleted or duplicated in at least two patients – 332 in ACC of which 305 were never impacted in controls, and 4 in CBLH each of which was also impacted in one control. Of the 305 ACC genes, 276 were from three well characterized callosal agenesis genomic regions: 8p22-p21.3, 8p23.3 and 1q41-qter (reviewed in [14]). These three regions had recurrent CNVs in our ACC patient population, which explains why most of the significantly enriched genes were from these regions. It also turned out that CNVs in these regions overlapped considerably (Figure 5 and Table 1). The remaining 29 genes (out of the 305) which were not from these three regions were all duplicated and found in small familial CNVs with low pathogenicity scores (discussed in a later section) or in CNVs that could not be independently confirmed. These 29 genes are indicated by asterisks in Table S3. The 4 genes in CBLH patients were from regions containing structural polymorphisms in normal individuals. Thus, our gene-based analysis did not reveal any novel genes enriched in patients relative to controls. This may be explained by a requirement for a much larger patient population for this type of analysis. However, as we show in the following sections, it is possible to identify some individual CNVs (as opposed to individual genes) that are likely to be deleterious even if they are found in a single patient by taking advantage of additional information such as inheritance of the CNV, its length, and phenotypic data from model organisms such as mouse.

**Table 1:** List of *de novo* and likely deleterious brain malformation CNVs ordered by their cytogenetic bands.

Patient ID	Malformation(s)	Cytoband	CNV type	Interval (Hg18)	Length (Mb)	Pathogenicity score	Known/novel
LR01-282	ACC-PMG	1p31.3-p31.1	del	chr1:61236413-75237180	14	19	Novel**
1412-0	ACC-CBLH-PMG	1p34.1	dup	chr1:45995673-46235468	0.24	9	Novel*
1574-0	ACC-CBLH	1q31.2-q31.3	del	chr1:190709879-193085819	2.38	15	Novel**
1574-0	ACC-CBLH	1q41-q42.13	del	chr1:220541565-228085645	7.54	20	Known
1187-0	ACC	1q43-q44	del	chr1:235378791-247169190	11.79	22	Known
1586-0	ACC-CBLH	1q43-q44	del	chr1:240394199-243529398	3.14	20	Known
1020-0	ACC-CBLH	1q44	dup	chr1:241945126-247185943	5.24	20	Known
LR02-049	ACC	2q11.2-q13	dup	chr2:96787353-113658240	16.87	22	Novel**
1070-0	ACC	2q22.2-q22.3	del	chr2:143970610-146955159	2.98	15	Known
1412-0	ACC-CBLH-PMG	4q13.3-q22.3	del	chr4:74621654-96253001	21.63	22	Known
LR08-130	CBLH	5q23.1	del	chr5:116416478-120055246	3.64	14	Novel**
1364-0	ACC	6q26-q27	del	chr6:161939434-170656616	8.72	21	Known
1546-0	ACC	7q35	del	chr7:142965893-143193381	0.23	10	Novel*
1300-0	ACC	7q36.3	dup	chr7:158396815-158558805	0.16	10	Novel*
1192-0	ACC	8p21.3-p11.1	dup	chr8:22609566-43689385	21.08	20	Known
1006-0	ACC	8p23.1-p11.1	dup	chr8:12595527-43811979	31.22	21	Known
1020-0	ACC-CBLH	8p23.1-p11.1	dup	chr8:12595527-43926760	31.33	22	Known
1228-0	ACC	8p23.1-p11.1	dup	chr8:12595527-43926760	31.33	22	Known
1356-0	ACC	8p23.1-p11.21	dup	chr8:12512547-42012236	29.5	20	Known
1041-0	ACC-CBLH	8p23.1-p12	dup	chr8:12595527-36223678	23.63	21	Known
1610-0	ACC	8p23.1-p12	dup	chr8:12538636-33311183	20.77	20	Known
1006-0	ACC	8p23.3-p23.1	del	chr8:183585-7808664	7.63	21	Known
1020-0	ACC-CBLH	8p23.3-p23.1	del	chr8:197819-6913279	6.72	22	Known
1041-0	ACC-CBLH	8p23.3-p23.1	del	chr8:179030-6913279	6.73	22	Known
1228-0	ACC	8p23.3-p23.1	del	chr8:179030-6913279	6.73	22	Known
1356-0	ACC	8p23.3-p23.1	del	chr8:154984-6913279	6.76	21	Known
1610-0	ACC	8q21.13-q21.3	del	chr8:82919646-88810045	5.89	18	Known
1127-0	ACC	9p23-p22.3	del	chr9:13034407-14653394	1.62	10	Novel
LR03-181	ACC	9q34.3	del	chr9:139238269-140156466	0.92	13	Known
1221-0	ACC-PMG	11p14.3-p14.2	dup	chr11:21871965-26730606	4.86	15	Novel**
1258-0	ACC	14q21.1	dup	chr14:38923501-39078057	0.15	10	Known
1020-0	ACC-CBLH	14q24.2	dup	chr14:69961223-70005628	0.04	7	Novel*
1130-0	ACC	15q11.2-q13.1	del	chr15:21172300-26208861	5.04	21	Novel**
1028-0	ACC	16p12.3	dup	chr16:18714812-18956879	0.24	9	Novel*
LP97-141a1	ACC-PMG	16p13.11	del	chr16:15369798-16195546	0.83	10	Novel
1351-0	ACC	17p13.3-p12	dup	chr17:15521-11307413	11.29	24	Known
1338-0	ACC	17q21.31	del	chr17:41063083-41562443	0.5	13	Known
1327-0	ACC	20p13	dup	chr20:391798-746755	0.35	13	Known
1223-0	ACC-CBLH	Xq28	dup	chrX:152829448-153034217	0.43	7	Known
LR02-148	CBLH	Xq28	dup	chrX:153072241-153534039	0.46	11	Known

“Known/Novel” refers to whether the CNV has been previously observed to be *de novo* in patients with one or more of the three brain malformations (Known) or not (Novel). While some of the “Novel” CNVs may previously have been implicated in other disorders, they are novel for the malformations used in this study. Double asterisks denote *de novo* CNVs at least 500 kb with high pathogenicity scores. Single asterisks denote *de novo* CNVs less than 500 kb with slightly lower pathogenicity scores. While deletions of 7q36.3 have been linked to ACC, duplications have not which is why it is “Novel”. Similarly, deletion 9p23-p22.3, which has not been observed in ACC, overlaps a known ACC region with duplications (see Table S9). Many *de novo* CNVs in ACC were found to overlap *de novo* CNVs observed in autism. All CNVs listed are *de novo* except for the following five: 9p23-p22.3, 9q34.3, Xq28 (1223-0) for which inheritance could not be determined; 16p13.11 which was paternally inherited and uncovered a maternally inherited nonsense pathogenic mutation in *NDE1*; and Xq28 (LR02-148) which was maternally inherited. While patient 1574-0 has one large *de novo* deletion on 1q41-q42.13 that overlaps a well-known ACC interval, the second *de novo* deletion on 1q31.2-q31.3 is also large and likely to be deleterious.

Pathway analysis using MetaCore from GeneGo Inc with genes from rare deletions that were at least 500 kilobases (kb) from ACC patients and controls showed a significant enrichment of genes involved in GABAergic synaptic transmission, GPCR signaling, and neurological signaling in patients (Table S4). This difference was still present when correcting for the larger CNVs present in the patient cohort.

### Independent Confirmation of Patient-specific CNVs

We found 250 patient-specific CNVs, which we defined as those that impacted less than 100% of the genes found in any given control CNV of the same copy state (see Materials and Methods). Unlike rare CNVs, these patient-specific CNVs were identified from patients of all ethnicities. We were able to independently confirm 199 (79.6%) CNVs by qPCR (Table S5). The remaining CNVs were false positives with most (36 of 51 or 70.6%) in the smaller 30–100 kb range. We were able to assess inheritance for 144 (72.4%) of the confirmed CNVs, of which 35 (24.3%) were *de novo*. Eighteen of these (51.4%) were deletions ranging from 227 kb to 21.63 Mb, and 17 (48.6%) were duplications ranging from 44 kb to 31.33 Mb. Twelve (34.3%) of the 35 *de novo* CNVs were submicroscopic (less than 4 Mb). Finally, 109 CNVs were inherited, representing 75.7% of those for which inheritance could be determined. A graphical summary of qPCR results is shown in Figure S7.

Of the 33 CNVs≥500 kb in ACC patients whose inheritance could be confirmed, 26 (78.8%) were *de novo* (Table S5). Thus, large CNVs are much more likely to be *de novo*. The proportions of ACC, ACC-PLUS, and ACC-ONLY patients with at least one *de novo* CNV were 9.4%, 6.9%, and 11.1%, respectively. In contrast, we found only one patient without ACC (this patient was diagnosed with CBLH only) who had a *de novo* CNV, in this case a 3.64 Mb deletion on chromosome 5q23.1. Table 1, which contains a subset of CNVs in Table S5, lists all *de novo* and other possibly pathogenic CNVs.

As *de novo* CNVs are likely to harbor causative brain malformation genes, we compiled a list of 1,318 genes found in *de novo* CNVs but not in any inherited patient or control CNVs of the same copy state. Coupled with mutations in mouse orthologs resulting in a nervous system phenotype, these serve as good candidates for novel human brain malformation genes (listed in Table S8).

Given the co-occurrence of ACC and autism in numerous prior reports [1], [2], [25]–[33], we asked whether CNVs observed in ACC and those in autism overlapped (that is, whether at least 50% of the genes in autism CNVs were also found in ACC CNVs). *De novo* CNVs in autism were compiled from several studies and are listed in Table S6 [34]–[39]. We found that 13 out of 34 (38.24%) *de novo* CNVs but only 5 out of 66 (7.58%) inherited CNVs in ACC patients overlapped with *de novo* CNVs reported in autism (p = 3.06×10^−4^; OR = 7.55; 95% CI = 2.40–23.72). The observed overlap may partly be explained by the co-occurrence of autism in our ACC cohort, as 47 of 172 (27.3%) ACC patients evaluated were diagnosed with this condition (Table S7), and partly by the presence of a shared genetic risk between ACC and autism.

### Novel Brain Malformation CNVs

We assigned each qPCR-validated CNV a pathogenicity score based on criteria such as size, inheritance, gene content, and overlap with known neurodevelopmental disorder or brain malformation CNVs (Table S5), similar to a scoring method that has been described previously [40]. All CNVs in Table S5 are sorted based on this pathogenicity score, with higher scores indicative of a higher likelihood of pathogenicity. Table 1, which is a subset of Table S5, lists all *de novo* and other likely deleterious CNVs addressed below.

Based on these rigorous criteria we found six novel *de novo* CNVs≥500 kb, one patient per CNV, with high pathogenicity scores in 1q31.2-q31.3 (ACC-CBLH deletion), 1p31.3-p31.1 (ACC-PMG deletion), 2q11.2-q13 (ACC-ONLY duplication), 5q23.1 (CBLH deletion), 11p14.3-p14.2 (ACC-PMG duplication), and 15q11.2-q13.1 (ACC-ONLY deletion). All of these were found in patients who had at least ACC with the exception of one CNV in a patient with isolated CBLH (the aforementioned 3.64 Mb deletion on 5q23.1). This CNV contains 10 genes including *HSD17B4*, mutations of which cause Perrault Syndrome with ataxia indicative of cerebellar involvement [41]. We also found five novel *de novo* CNVs less than 500 kb with slightly lower pathogenicity scores. Three of these were in 7q35 (ACC-ONLY deletion), 7q36.3 (ACC-ONLY duplication), and 16p12.3 (ACC-ONLY duplication). The other two were in 1p34.1 (ACC-CBLH-PMG duplication) and 14q24.2 (ACC-CBLH duplication), although other much larger *de novo* CNVs previously implicated in brain malformations were found in patients with these two particular CNVs as well (Table 1).

Within the previously defined ACC region 7q36, we identified a *de novo* 162 kb duplication in a patient with complete ACC and subcortical heterotopias (Table 1). One of the genes in this interval is *VIPR2*, duplications of which have recently been implicated in schizophrenia [42], [43]. Note that deletion, but not duplication, of 7q36 has previously been reported in patients with ACC [14].

We next individually examined the remaining qPCR-confirmed CNVs that were either inherited or had unknown inheritance because parental DNA samples were not available and found 4 more that are likely deleterious based on their gene content and genomic location (Table 1). The first two were overlapping duplications in Xq28 in males with CBLH (one of who also had ACC) that included the *FLNA* gene associated with periventricular nodular heterotopias [44]. One of the males had a duplication of *MECP2*, which has been associated with severe developmental handicaps in boys [45]. This region has been linked to several different phenotypes, so that studies in additional patients will be needed to define the critical region. The third was a 1.6 Mb deletion of 9p23-p22.3 which impacts *NFIB*, a gene that causes callosal defects in both homozygous and heterozygous mouse mutants [46]. Incidentally, in another patient we also identified a *de novo* deletion at chromosome 1p31.3-p31.1 that included *NFIA*, another nuclear factor-I family member also associated with ACC in both patients and animal models [47], [48].

Finally, the fourth CNV was a paternally inherited 0.83 Mb deletion in a patient with ACC-PMG on 16p13.11, a known risk factor for autism, intellectual disability, schizophrenia, and epilepsy [49], [50]. Brain imaging demonstrated extreme microcephaly with very low forehead, enlarged extra-axial fluid, complex polymicrogyria-like cortical malformation and complete ACC. We hypothesized that his phenotype was too severe to be caused by just a single allele deletion of 16p13.11, and therefore carried out whole exome sequencing. This led to the identification of a novel exonic chr16:15761189 C/T (hg19) maternally inherited nonsense mutation (p.R44X) in the non-deleted allele of *NDE1*, a gene located within the 16p13.11 interval. Figure S8 shows Sanger sequencing-based confirmation of this mutation. Thus, the deletion uncovered a loss of function allele of *NDE1*, resulting in an autosomal recessive phenotype resembling the severe microcephaly syndrome associated with homozygous mutations of *NDE1* [51], [52]. We concurrently published a separate detailed study involving this particular patient shortly after this finding from the current CNV study [53].

### Refining Previously Known Pathogenic CNVs

We determined whether any of the CNVs confirmed by qPCR mapped to previously known cytogenetic regions associated with ACC [14], CBLH (literature search), and PMG [4] in order to confirm and narrow the critical regions and/or eliminate regions unlikely to contribute to disease especially if they overlapped with small inherited CNVs. Of 50 known ACC intervals, 30 had one or more ACC patients from our cohort with at least one overlapping CNV, and 10 of these overlapped with at least one *de novo* CNV, thereby providing additional information regarding the critical interval (Table S9) and also confirming previous findings. One *de novo* CNV overlapped a known PMG interval on 4q21.21–q22.1 and another overlapped the 17q21.31 deletion syndrome, which has only recently been associated with ACC [54]. Haploinsufficiency of *KANSL1* was recently shown to be the likely cause of this syndrome [55], [56].

Finally, we found 5 patients in our current cohort with the well-known 8p inverted duplication deletion syndrome, making this the most common recurrent genomic region in ACC patients (5/255 or 2%). Importantly, these patients were recruited specifically for our current CNV study with no prior ascertainment bias for a chromosomal abnormality. We previously observed this recurrent event at a rate of 0.8% (4/472) in a California birth cohort of children with callosal agenesis [3]. In our present study we also identified two individuals with ACC, microcephaly and other features common to the larger genomic rearrangement who had small 8p duplications (but no 8p deletions) contained within the larger duplicated interval identified previously. These two duplications define a narrowed critical interval and point toward the duplication as the causative CNV (Figure 5 and Table S10).

## Discussion

This is the first large-scale CNV analysis involving common brain malformations. Our investigation has demonstrated that large CNVs, particularly deletions, are highly enriched in this well-defined ACC cohort, and our study has led to significant narrowing of critical regions for several ACC chromosomal intervals and discovery of six novel CNVs (five ACC and one CBLH) with high pathogenicity scores. These discoveries will facilitate both the interpretation of CNV results in patients with many developmental disorders and the discovery of the underlying genes and pathways.

The genomic burden of large, gene-rich, rare CNVs, particularly deletions, was significantly higher in ACC, ACC-ONLY, and ACC-PLUS patients than controls. Unexpectedly, the burden of rare CNVs was not significantly different in patients with CBLH or PMG (with or without ACC) than in controls. While several causative CNVs have been identified for these malformations [4], [15]–[23], our large-scale study suggests that CNVs may not explain a significant proportion of patients with these malformations. These differences may in part be explained by extrinsic (non-genetic) causes that may be more important for CBLH and PMG than for ACC and other developmental brain disorders [57]–[59]. We also have not addressed (except in the case of *NDE1*) single gene autosomal recessive models or oligogenic recessive models, which could explain the causes for some or many of the CBLH and PMG cases in our cohort. Therefore, our hypothesis that the genomic burden of rare genic CNVs will be significantly higher in patients compared to controls was supported for ACC but not for the other two malformations, and thus further genetic investigation is warranted for all three groups of disorders.

The hypothesis that there would be an enrichment of large *de novo* CNVs in the patient cohort was also supported for ACC but not CBLH or PMG as we observed that 9.4% of ACC patients had at least one *de novo* CNV, with 7.1% having at least one large *de novo* CNV≥500 kb. As we had anticipated, almost one-third (34.5%) of all *de novo* CNVs were sub-microscopic (less than 4 Mb).

Intriguingly, a significant number of *de novo* but not inherited CNVs in ACC overlapped *de novo* CNVs previously observed in patients with autism. One explanation for this is that our ACC cohort was skewed towards patients who had a developmental disability, including autism, since this was the reason why many of these patients were initially ascertained. From Table S7, which lists four features of developmental disability in all of our ACC patients, only 16 (15.8%) patients (double asterisks) out of the 101 patients (single asterisks) in whom all four features were evaluated had no developmental impairment. None of the CNVs from these 16 patients overlapped *de novo* CNVs in autism. Thus, because all ACC patients whose *de novo* CNVs overlapped *de novo* autism CNVs had some form of developmental disability, their *de novo* CNVs may contribute less towards ACC and more towards the developmental disability. Another explanation is that the observed overlap points to a shared genetic risk between ACC and autism for the following reasons. First, it has been shown that there is an overlapping genetic risk among divergent neurological disorders indicating that, while there may be some unique variants for each class of disorder, there are many that are also common [60]–[63]. This may be due to these neurological disorders (including ACC and autism) being complex, genetically heterogeneous with variable expressivity, and often caused by disruption of multiple genes each of which has a small to moderate effect. Second, a plethora of studies, including an extensive meta-analysis [64], has demonstrated unequivocally that the corpus callosum area is reduced and that its structure and integrity are compromised in patients with autism [25]–[33]. This is in keeping with findings of under-connectivity of functional brain networks and aberrant interhemispheric information transfer in autism patients [65], [66] and in a mouse model [67]. Third, the vast majority of patients with ACC have disruption of higher order cognition, social intelligence, or both. They have variable deficits in social and executive functioning as shown by several studies of high functioning ACC individuals with IQ in the normal range [1], [2], [68]. While these observations are suggestive, additional studies including single gene analyses will be needed to address more directly whether ACC and autism indeed have a shared genetic basis.

Our study demonstrates a prevalence of large gene-rich *de novo* CNVs in ACC but not in CBLH or PMG. Additional patients with overlapping CNVs as well as mutations in candidate genes identified by deep sequencing will be needed to identify one or more novel causal genes with major effects, which can then be followed up with studies in model organisms. In addition, patients who did not have any likely pathogenic CNVs will have to be subjected to additional analyses such as exome sequencing and assessing non-genic regions that may disrupt the regulation of causative genes.

## Materials and Methods

### Ethics Statement

Informed consent was obtained from all subjects, and the study was approved by the institutional review boards at participating institutions: the University of Chicago, Seattle Children's Hospital, and the University of California at San Francisco.

### Patient Population

All subjects were recruited with informed consent for participation in the study. We genotyped 545 patients aged 0–60's on high-throughput genome-wide SNP arrays using the InfiniumII HumanHap610 BeadChip technology (Illumina San Diego CA), at the Center for Applied Genomics at Children's Hospital of Philadelphia (CHOP). Diagnosis was done by systematically reviewing magnetic resonance imaging (MRI) brain scans to include a comprehensive assessment of prosencephalic, mesencephalic, and metencephalic structures. Fifteen percent of patients had a previous cytogenetic and/or BAC array-CGH performed with no abnormal findings. Genomic DNA for the vast majority of patients was extracted from whole blood or lymphoblast cells, and for others from saliva using standard protocols. About 1 ug of non-degraded DNA samples with A260/280 ratios ≥1.8 were used for array genotyping.

### Control Population

2,349 controls (1,953 Caucasian; 160 Asian; 236 African based on self-reports and confirmed by genotyping) were genotyped on the same platform as the patients. Subjects were primarily recruited from the Philadelphia region through the Hospital's Health Care Network and included disease-free children in the age range of 0–18 yrs of age who had high quality, genome-wide genotyping data from blood samples, and no serious underlying medical disorders, including but not limited to neurodevelopmental disorders, cancer, chromosomal abnormalities, and known metabolic or genetic disorders.

### Array Quality Control

Only samples with a SNP call rate >98% and a standard deviation (SD) of the Log R Ratio (LRR)<0.30 were included. Only samples whose GC wave factor (GCWF) of LRR ranged between −0.04<X<0.04 were accepted, as those with values outside of this range show wave artifacts roughly correlating with GC content and which are known to interfere with accurate calling of CNVs. If the count of CNV calls made by PennCNV exceeded 100, the DNA quality was usually poor. Thus, only samples with CNV call count <100 were included. For any related samples (such as siblings) only one sample was included. Ethnicities were determined based on Eigenstrat scoring.

### Identification of CNVs

CNVs were identified using PennCNV [24] which combined multiple sources of information, including Log R Ratio and B Allele Frequency at each SNP marker, along with SNP spacing, a trained hidden Markov model, and population frequency of the B allele to generate CNV calls. The following criteria were used for selecting CNVs for downstream analyses: (i) presence of at least 10 contiguous SNPs, (ii) a length of at least 30 kb, (iii) a PennCNV confidence score of at least 10, and (iv) impacting at least one exon, including the untranslated regions of a gene. The resulting CNVs from every individual were then merged if they were less than 50 kb apart or up to 200 kb apart if at least one of the CNV was over 1 Mb. This set of filtered CNV, referred to as “CNV List 1” from here on, was used to identify rare CNVs based on the presence of at least one gene impacted at a frequency of less than 1% in the combined population of unrelated Caucasian controls and patients in a particular malformation group (ACC, ACC-PLUS, ACC-ONLY, CBLH, and PMG). We first identified rare deletions and duplications together from an initial input of deletions and duplications found in CNV List 1. Then we identified rare deletions and duplications separately from an input of deletions only and duplications only, respectively, from CNV List 1.

### Power Analysis

We used G*Power 3.1.6 [69] to calculate the level of power achieved for detecting significant differences in the burden of rare CNVs that were at least 1 Mb using our patient and control population sizes. The following settings were used: Test family = Exact, Statistical test = Proportions: Inequality, two independent groups (Fisher's Exact Test), Type of power analysis = Post hoc: Compute achieved power – given alpha, sample size, and effect size. Alpha was set to be 0.05. Sample sizes were the numbers of ACC, CBLH, PMG patients (group 1) and controls (group 2). Effect size was set to be the proportion of ACC patients (proportion 1) and controls (proportion 2) with rare CNVs≥1 Mb. Two tails were used when computing power for ACC and one tail when computing power for CBLH and PMG. This is because it had to be assumed that patients with the latter two disorders, and not controls, were enriched with rare CNVs≥1 Mb similar to ACC patients.

### Genome-Wide Burden of Rare CNVs

We assessed the genome-wide burden of rare CNVs in each malformation group as well as in controls based on three different measures. The first measure was based on CNV size (all CNVs≥30 kb, CNVs≥1 Mb, 1 Mb>CNVs≥500 kb, 500 kb>CNVs≥100 kb, and 100 kb>CNVs≥30 kb). Note that the first category comprises all rare CNVs. The second measure was based on the number of genes with at least one exon impacted by rare CNVs (as determined by Hg18 coordinates on the UCSC Genome Browser under the ‘UCSC Genes’ track). Each of the five CNV size classes above was further divided into five sub-classes based on gene number (CNVs with 1 gene, 2–5 genes, 6–10 genes, 11–20 genes, and more than 20 genes). The third measure of assessing rare CNV burden was based on the number of CNVs per genome in patients and controls. Specifically, each of the five size classes above were divided into six sub-classes based on their number per genome (≥1 CNVs, exactly 1 CNV, 2 CNVs, 3 CNVs, 4 CNVs, and ≥5 CNVs). In each sub-class, we first assessed the burden of deletions and duplications together, followed by one or the other separately. The total number of tests done per size class were therefore 33 (Table S2).

### Impact Frequencies of Individual Genes from Rare CNVs

For each malformation, genes were compiled from three lists of rare CNVs (deletions and duplications together, and each separately). From a given CNV we only assessed those that were impacted at a frequency of less than 1% (i.e. rare genes) in the combined population of patients and controls. The numbers of patients and controls in whom a gene was impacted were counted and significant differences determined by two-tailed Fisher's Exact Test. Multiple testing was carried out using the Benjamini-Hochberg method.

### Identifying Patient-Specific CNVs for Independent Confirmation

We applied two additional filtering criteria on CNVs found in CNV List 1 (see “Identification of CNVs” section above) in order to prioritize the discovery of patient-specific CNVs that are more likely to be pathogenic. A patient CNV was considered a good candidate for independent confirmation and inheritance evaluation if (i) it impacted at least one gene not impacted by a given control CNV and (ii) if its copy state was different from that of a given control CNV even if the gene content was identical. Because the overlap between patient and control CNVs was gene-based, these CNVs did not have to physically overlap. To measure the gene-based overlap, we first determined the number of controls with at least one CNV that shared one or more genes found in a given patient CNV. The control CNV sharing the highest number of genes with a patient CNV was then used to determine the cutoff. For example, if 10 genes were deleted in a single patient and each one of those genes was deleted individually in 10 different control samples, then the patient CNV was still considered a good candidate for inheritance evaluation (since the highest overlap with a given control CNV in this case is only 10%). However, if all 10 genes were deleted in a single control individual, whether by one contiguous CNV or several CNVs that could not be merged based on the merging criteria mentioned previously, then the patient CNV was not considered patient-specific. From the list of patient CNVs that satisfied these criteria, we removed those that were highly recurrent in both patients and controls in order to eliminate regions that are highly polymorphic and not likely to be pathogenic even if the gene-based overlap was less than 100%. Examples of such regions include 14q11.2, 14q32.33, and Xq21.31. This resulted in 250 patient-specific CNVs.

### Independent Confirmation and Inheritance Evaluation of Patient-Specific CNVs by Quantitative PCR (qPCR)

We used Power SYBR Green PCR Master Mix (Applied Biosystems) to both confirm patient CNVs and evaluate their inheritance using parental DNA when available. At least two independent primer pairs were used for each CNV that was tested. Quantitative PCR was carried out on an ABI 7900 (Applied Biosystems) machine in 384-well plates using either the SDS 2.2 or 2.3 software. All PCR products were in the range of 90–160 bp. Each sample was performed in triplicate in a total reaction volume of 10 ul using 20 ng of DNA and 300 nM final concentration of each primer. At least two (one male and one female) and no more than four (two males and two females) unrelated normal control individuals were also run alongside the patient-parent trio for each CNV that was tested. PCR conditions were 95°C denaturation for 10 minutes; 40 cycles each of 95°C for 15 seconds and 60°C for 1 minute; a dissociation curve at 95°C for 15 seconds, 60°C for 15 seconds, and again 95°C for 15 seconds. The passive internal reference dye (ROX) was used to adjust for any differences in the final reaction volumes across multiple wells. Data analysis was carried out using the ΔΔCT method to obtain the relative quantity (RQ) values after normalizing with at least two of four reference genes (*ALB*, *RPP14*, *HEM3*, and *GPR15*). Thus, for each sample at least 8 different RQ values were obtained (2 different primers×2 different normal control individuals×2 different reference genes). If the 95% confidence interval of the mean RQ was between 0.7 and 1.3 then the copy state was considered to be normal. Values less than 0.7 or higher than 1.3 were considered deletions or duplications, respectively. Primers were designed using Primer3 (http://frodo.wi.mit.edu/primer3/).

### Overlap between CNVs Observed in ACC and CNVs in Autism

We compiled a list of 231 (138 deletions and 93 duplications) exon-impacting *de novo* CNVs observed in autism. A CNV in ACC was considered to overlap with a CNV in autism if it contained at least 50% of the genes in the latter.

### Whole Exome Sequencing

We performed exome sequencing on DNA from patient LP09-141a1 (ACC-PMG) with a paternally inherited deletion on 16p13.11. We used the Agilent SureSelect 50 Mb All Exon kit for target capture, and sequenced 100 base pair paired end reads on Illumina Hiseq. Exome data was aligned to hg19 with Burroughs-Wheeler aligner (http://bio-bwa.sourceforge.net/bwa.shtml). Duplicate reads were marked using Picard (http://picard.sourceforge.net/) and excluded from further analysis. Single nucleotide variants (SNVs) and short insertions and deletions (indels) were called using samtools and exome coverage was determined with Genome Analysis Toolkit (http://www.broadinstitute.org/gsa/wiki/index.php/The_Genome_Analysis_Toolkit). Variants were annotated using SeattleSeq (http://snp.gs.washington.edu/SeattleSeqAnnotation/) and custom scripts identified variants affecting coding sequence. Novel variants were identified by filtering against >2500 publicly available exomes in the Exome Variant Server (http://evs.gs.washington.edu/EVS/). Nonsynonymous variants were validated by Sanger sequencing of DNA in proband and both parents as per routine protocol.

### Statistical Tests

All significant differences were determined by calculating a two-tailed Fisher's Exact Test p-value and then correcting for multiple testing using the Bonferroni method. For rare CNV burden analysis the Fisher's p-value for each test in a given CNV size class was multiplied by 33 as this was the total number of tests carried out per size class. A corrected p-value of 0.05 or smaller was considered significant. The odds ratios and their 95% confidence intervals were also calculated. For impact frequencies of individual genes in rare CNVs we used the Benjamini-Hochberg method of multiple testing correction to determine false discovery rates.

## Supporting Information

Figure S1 Power analysis plot showing the achieved power as a function of sample (population) size for detecting a significant enrichment of rare CNVs≥1 Mb in patients than controls. The x-axis shows the combined population of patients and controls just under the vertical marks. Just below this are the actual numbers of patients and controls in italic text (patients/controls) for ACC (top row), CBLH (middle row) and PMG (bottom row). Our analysis showed a significant enrichment of rare CNVs≥1 Mb in ACC but not in CBLH or PMG patients. The appropriately colored lines intersecting the curves for the three malformations indicate the power level we achieved using our population sizes. For CBLH and PMG these lines represent the power level we would have achieved had there been an enrichment of rare CNVs≥1 Mb in these patients similar to that in ACC patients, and show that our patient population was sufficiently large since all power levels are greater than 0.8.
(TIF)

Figure S2 Genome-wide burden of rare CNVs of various sub-classes based on CNV size, number of CNVs per genome, and number of exonic genes impacted in 205 ACC patients and 1,953 controls of Caucasian ethnicity. Deletions and duplications analyzed together are shown by “±”, deletions analyzed separately by “−”, and duplications analyzed separately by “+”. Panel A shows sub-categories based on number of genes and number of CNVs per genome for all rare CNVs≥30 kb regardless of size. Panels B–E show these same sub-categories for various size classes of rare CNVs. These size classes are: rare CNVs that were at least 1 Mb (≥1 Mb; panel B), those that were at least 500 kb but less than 1 Mb (500 kb–1 Mb; panel C), those that were at least 100 kb but less than 500 kb (100–500 kb; panel D), and those that were at least 30 kb but less than 100 kb (30–100 kb; panel E). Significant differences between patients (dark bars) and controls (light bars) are shown by black lines/hooks that connect patients and controls with numbers listed above. The numbers correspond to corrected p-values, odds ratios (OR), and 95% confidence intervals (CI) provided in the lower right. Asterisk: while the corrected p-value was not significant (0.27), the odds ratio (3.42) and 95% confident interval (1.43–8.18) were both highly suggestive of a significant difference.
(TIF)

Figure S3 Genome-wide burden of rare CNVs of various sub-classes based on CNV size, number of exonic genes impacted, and number of CNVs per genome in 82 ACC-PLUS patients and 1,953 controls of Caucasian ethnicity. Deletions and duplications analyzed together are shown by “±”, deletions analyzed separately by “−”, and duplications analyzed separately by “+”. Panel A shows sub-categories based on number of genes and number of CNVs per genome for all rare CNVs≥30 kb regardless of size. Panels B–E show these same sub-categories for various size classes of rare CNVs. These size classes are: rare CNVs that were at least 1 Mb (≥1 Mb; panel B), those that were at least 500 kb but less than 1 Mb (500 kb–1 Mb; panel C), those that were at least 100 kb but less than 500 kb (100–500 kb; panel D), and those that were at least 30 kb but less than 100 kb (30–100 kb; panel E). Significant differences between patients (dark bars) and controls (light bars) are shown by black lines/hooks that connect patients and controls with numbers listed above. The numbers correspond to corrected p-values, odds ratios (OR), and 95% confidence intervals (CI) provided in the lower right. Asterisk: while the corrected p-value was not significant (0.29), the odds ratio (3.16) and 95% confident interval (1.39–7.17) were both highly suggestive of a significant difference.
(TIF)

Figure S4 Genome-wide burden of rare CNVs of various sub-classes based on CNV size, number of exonic genes impacted, and number of CNVs per genome in 121 ACC-ONLY patients and 1,953 controls of Caucasian ethnicity. Deletions and duplications analyzed together are shown by “±”, deletions analyzed separately by “−”, and duplications analyzed separately by “+”. Panel A shows sub-categories based on number of genes and number of CNVs per genome for all rare CNVs≥30 kb regardless of size. Panels B–E show these same sub-categories for various size classes of rare CNVs. These size classes are: rare CNVs that were at least 1 Mb (≥1 Mb; panel B), those that were at least 500 kb but less than 1 Mb (500 kb–1 Mb; panel C), those that were at least 100 kb but less than 500 kb (100–500 kb; panel D), and those that were at least 30 kb but less than 100 kb (30–100 kb; panel E). Significant differences between patients (dark bars) and controls (light bars) are shown by black lines/hooks that connect patients and controls with numbers listed above. The numbers correspond to corrected p-values, odds ratios (OR), and 95% confidence intervals (CI) provided in the lower right. Asterisk: while the corrected p-value was not significant (1.00), the odds ratio (2.36) and 95% confident interval (1.10–5.06) were both highly suggestive of a significant difference.
(TIF)

Figure S5 Genome-wide burden of rare CNVs of various sub-classes based on CNV size, number of exonic genes impacted, and number of CNVs per genome in 180 CBLH patients and 1,953 controls of Caucasian ethnicity. No significant differences were observed between patients and controls in any CNV category analyzed. Deletions and duplications analyzed together are shown by “±”, deletions analyzed separately by “−”, and duplications analyzed separately by “+”. Panel A shows sub-categories based on number of genes and number of CNVs per genome for all rare CNVs≥30 kb regardless of size. Panels B–E show these same sub-categories for various size classes of rare CNVs. These size classes are: rare CNVs that were at least 1 Mb (≥1 Mb; panel B), those that were at least 500 kb but less than 1 Mb (500 kb–1 Mb; panel C), those that were at least 100 kb but less than 500 kb (100–500 kb; panel D), and those that were at least 30 kb but less than 100 kb (30–100 kb; panel E).
(TIF)

Figure S6 Genome-wide burden of rare CNVs of various sub-classes based on CNV size, number of exonic genes impacted, and number of CNVs per genome in 121 PMG patients and 1,953 controls of Caucasian ethnicity. No significant differences were observed between patients and controls in any CNV category analyzed. Deletions and duplications analyzed together are shown by “±”, deletions analyzed separately by “−”, and duplications analyzed separately by “+”. Panel A shows sub-categories based on number of genes and number of CNVs per genome for all rare CNVs≥30 kb regardless of size. Panels B–E show these same sub-categories for various size classes of rare CNVs. These size classes are: rare CNVs that were at least 1 Mb (≥1 Mb; panel B), those that were at least 500 kb but less than 1 Mb (500 kb–1 Mb; panel C), those that were at least 100 kb but less than 500 kb (100–500 kb; panel D), and those that were at least 30 kb but less than 100 kb (30–100 kb; panel E).
(TIF)

Figure S7 Summary of qPCR results showing the percent of all 250 patient-specific CNVs selected for independent confirmation. CNVs were classified as *de novo*, inherited (Inh), inheritance not determined (ND), and false positives on array (FP). These proportions are also provided for CNVs of different size classes, showing that smaller CNVs are more likely to be false positive array results. These size classes are: all patient-specific CNVs selected for qPCR confirmation that were at least 30 kb (≥30 kb), those that were at least 1 Mb (≥1 Mb), those that were at least 500 kb but less than 1 Mb (500 kb–1 Mb), those that were at least 100 kb but less than 500 kb (100–500 kb), and those that were at least 30 kb but less than 100 kb (30–100 kb). Table S5 provides a detailed listing of each CNV tested by qPCR.
(TIF)

Figure S8 Sequence chromatogram of the non-deleted allele of NDE1 in a male patient (ID: LP97-141a1) diagnosed with ACC-PMG showing a maternally inherited nonsense p.R44X mutation at chr16:15761189 C/T (Hg19) shown by the arrow.
(TIF)

Table S1 List of all rare CNVs in patients with ACC, ACC-PLUS, ACC-ONLY, CBLH, and PMG.
(XLS)

Table S2 Genome-wide rare CNV burden analysis in patients with ACC, ACC-PLUS, ACC-ONLY, CBLH, and PMG.
(XLS)

Table S3 List of rare genes significantly over-represented (FDR no more than 0.05) in ACC and CBLH patients.
(XLS)

Table S4 Pathway analysis using genes from rare CNVs at least 500 kb in ACC patients and controls.
(XLS)

Table S5 Independent qPCR-based confirmation of patient-specific CNVs and their inheritance.
(XLS)

Table S6 List of *de novo* CNVs observed in autism.
(XLS)

Table S7 Clinical features of all 255 ACC patients with good array quality control scores that were used in this study.
(XLS)

Table S8 Genes found only in *de novo* but not in inherited patient CNVs or in any control CNVs of the same copy state.
(XLS)

Table S9 Physical overlap of previously known large cytogenetic brain malformation regions with brain malformation CNVs found in this current study.
(XLS)

Table S10 Clinical features of patients with 8p CNVs.
(XLS)
