# Supplementary figures and images for: Both Rare and De Novo Copy Number Variants Are Prevalent in Agenesis of the Corpus Callosum but Not in Cerebellar Hypoplasia or Polymicrogyria
Source: PLoS Genet. 2013 Oct 3;9(10):e1003823. doi: 10.1371/journal.pgen.1003823 (PMC3789824; doi:10.1371/journal.pgen.1003823)

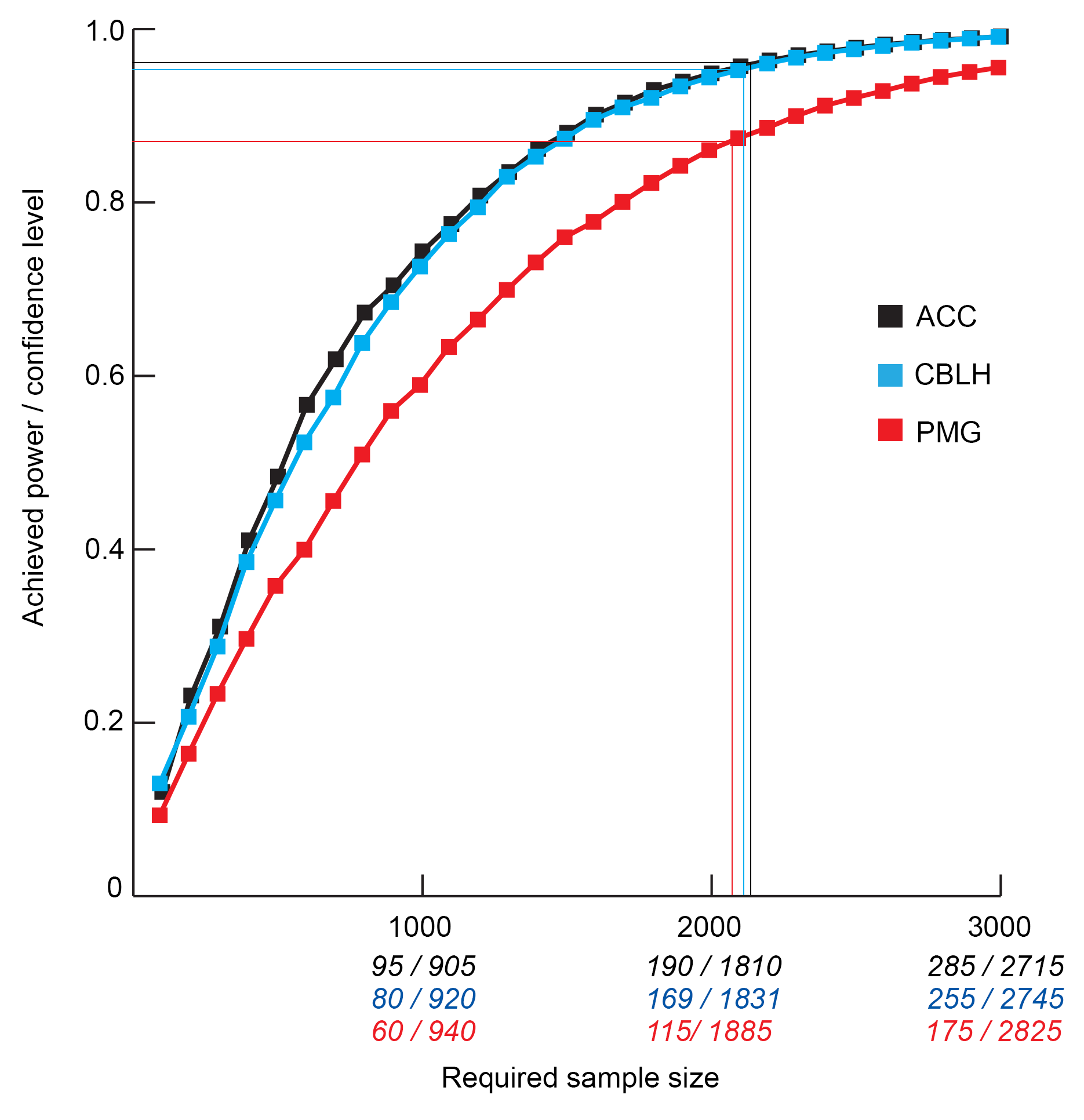

Supplement: Figure S1 — Power analysis plot showing the achieved power as a function of sample (population) size for detecting a significant enrichment of rare CNVs≥1 Mb in patients than controls. The x-axis shows the combined population of patients and controls just under the vertical marks. Just below this are the actual numbers of patients and controls in italic text (patients/controls) for ACC (top row), CBLH (middle row) and PMG (bottom row). Our analysis showed a significant enrichment of rare CNVs≥1 Mb in ACC but not in CBLH or PMG patients. The appropriately colored lines intersecting the curves for the three malformations indicate the power level we achieved using our population sizes. For CBLH and PMG these lines represent the power level we would have achieved had there been an enrichment of rare CNVs≥1 Mb in these patients similar to that in ACC patients, and show that our patient population was sufficiently large since all power levels are greater than 0.8. (TIF) [file pgen.1003823.s001.tif]

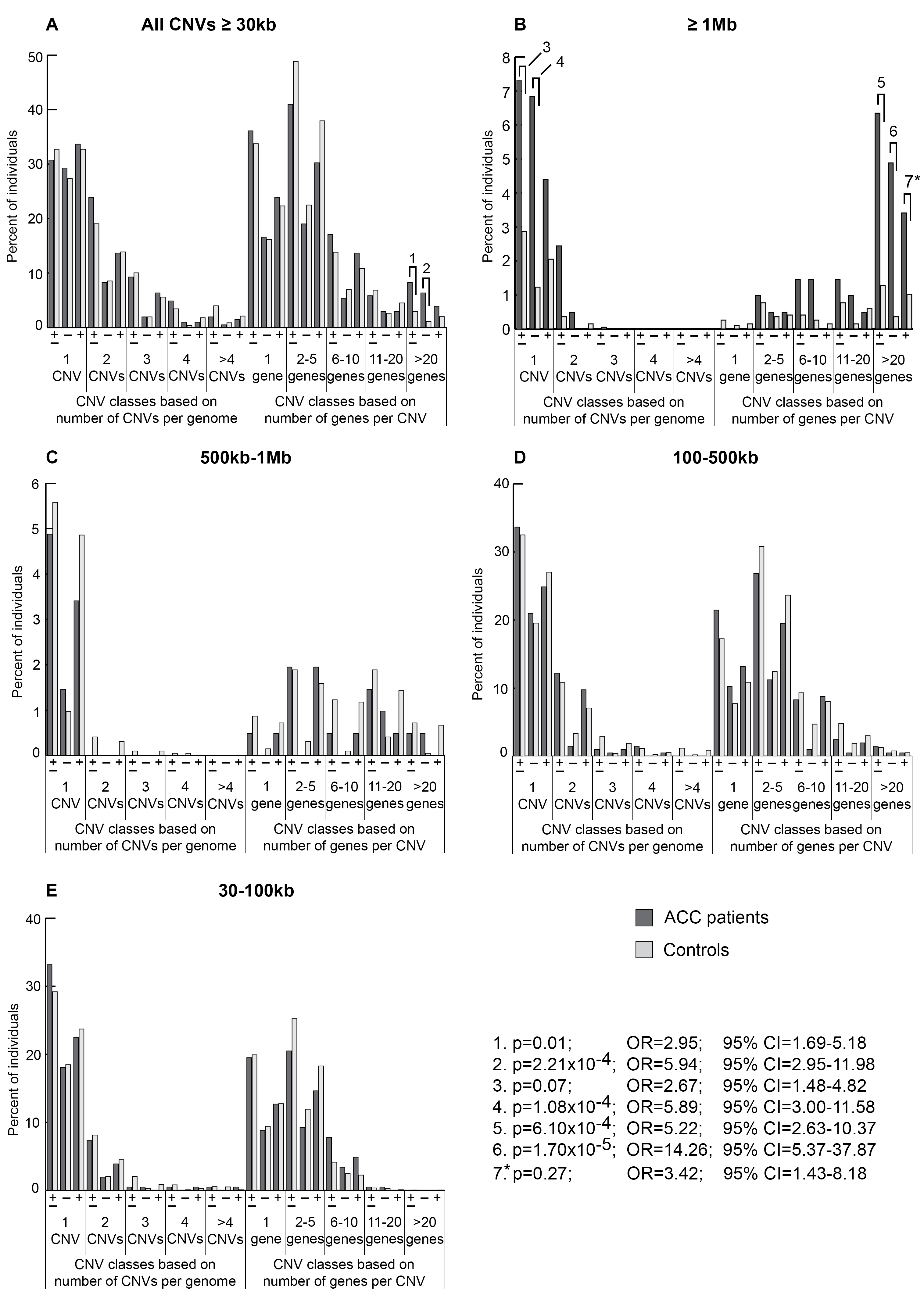

Supplement: Figure S2 — Genome-wide burden of rare CNVs of various sub-classes based on CNV size, number of CNVs per genome, and number of exonic genes impacted in 205 ACC patients and 1,953 controls of Caucasian ethnicity. Deletions and duplications analyzed together are shown by “±”, deletions analyzed separately by “−”, and duplications analyzed separately by “+”. Panel A shows sub-categories based on number of genes and number of CNVs per genome for all rare CNVs≥30 kb regardless of size. Panels B–E show these same sub-categories for various size classes of rare CNVs. These size classes are: rare CNVs that were at least 1 Mb (≥1 Mb; panel B), those that were at least 500 kb but less than 1 Mb (500 kb–1 Mb; panel C), those that were at least 100 kb but less than 500 kb (100–500 kb; panel D), and those that were at least 30 kb but less than 100 kb (30–100 kb; panel E). Significant differences between patients (dark bars) and controls (light bars) are shown by black lines/hooks that connect patients and controls with numbers listed above. The numbers correspond to corrected p-values, odds ratios (OR), and 95% confidence intervals (CI) provided in the lower right. Asterisk: while the corrected p-value was not significant (0.27), the odds ratio (3.42) and 95% confident interval (1.43–8.18) were both highly suggestive of a significant difference. (TIF) [file pgen.1003823.s002.tif]

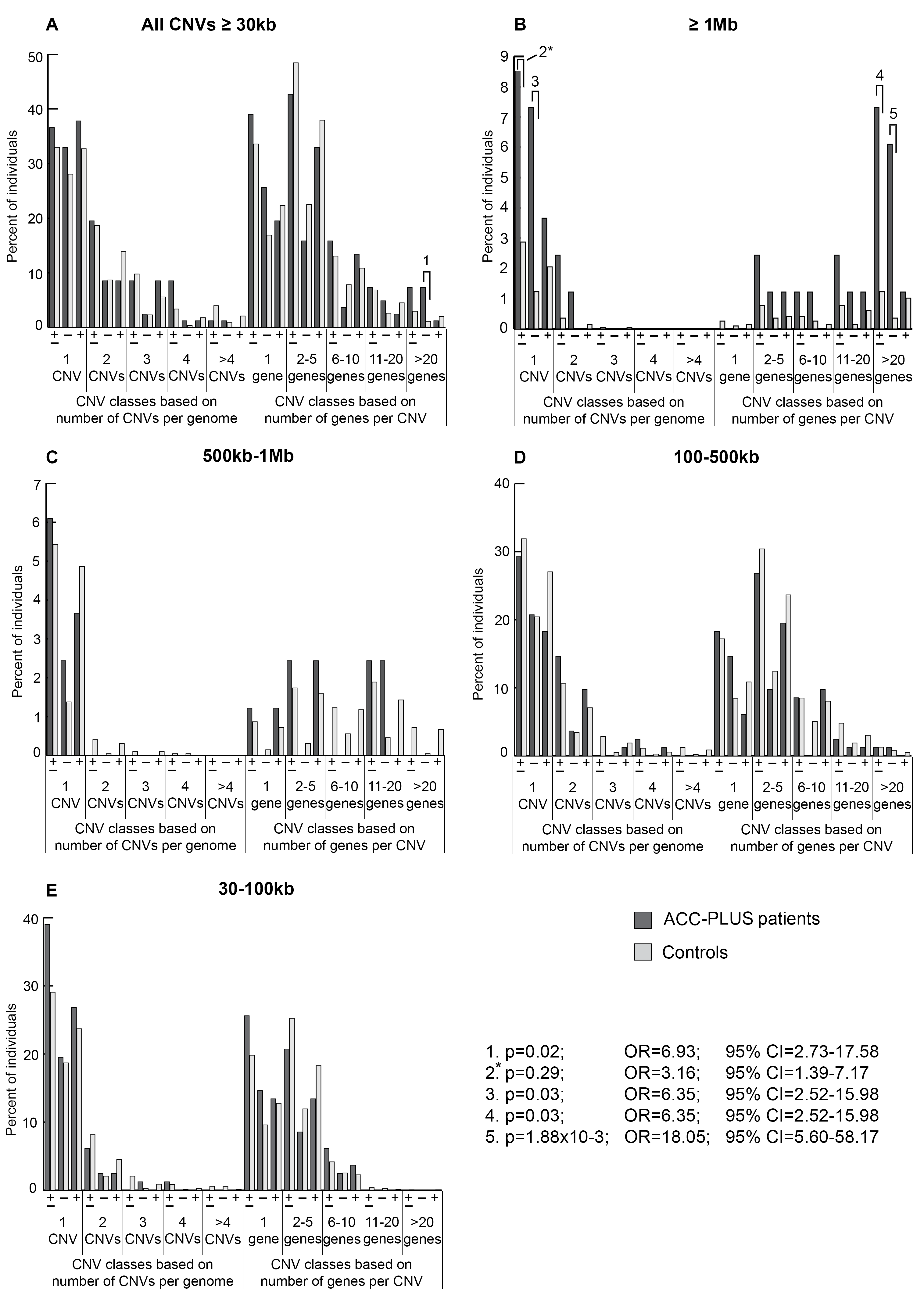

Supplement: Figure S3 — Genome-wide burden of rare CNVs of various sub-classes based on CNV size, number of exonic genes impacted, and number of CNVs per genome in 82 ACC-PLUS patients and 1,953 controls of Caucasian ethnicity. Deletions and duplications analyzed together are shown by “±”, deletions analyzed separately by “−”, and duplications analyzed separately by “+”. Panel A shows sub-categories based on number of genes and number of CNVs per genome for all rare CNVs≥30 kb regardless of size. Panels B–E show these same sub-categories for various size classes of rare CNVs. These size classes are: rare CNVs that were at least 1 Mb (≥1 Mb; panel B), those that were at least 500 kb but less than 1 Mb (500 kb–1 Mb; panel C), those that were at least 100 kb but less than 500 kb (100–500 kb; panel D), and those that were at least 30 kb but less than 100 kb (30–100 kb; panel E). Significant differences between patients (dark bars) and controls (light bars) are shown by black lines/hooks that connect patients and controls with numbers listed above. The numbers correspond to corrected p-values, odds ratios (OR), and 95% confidence intervals (CI) provided in the lower right. Asterisk: while the corrected p-value was not significant (0.29), the odds ratio (3.16) and 95% confident interval (1.39–7.17) were both highly suggestive of a significant difference. (TIF) [file pgen.1003823.s003.tif]

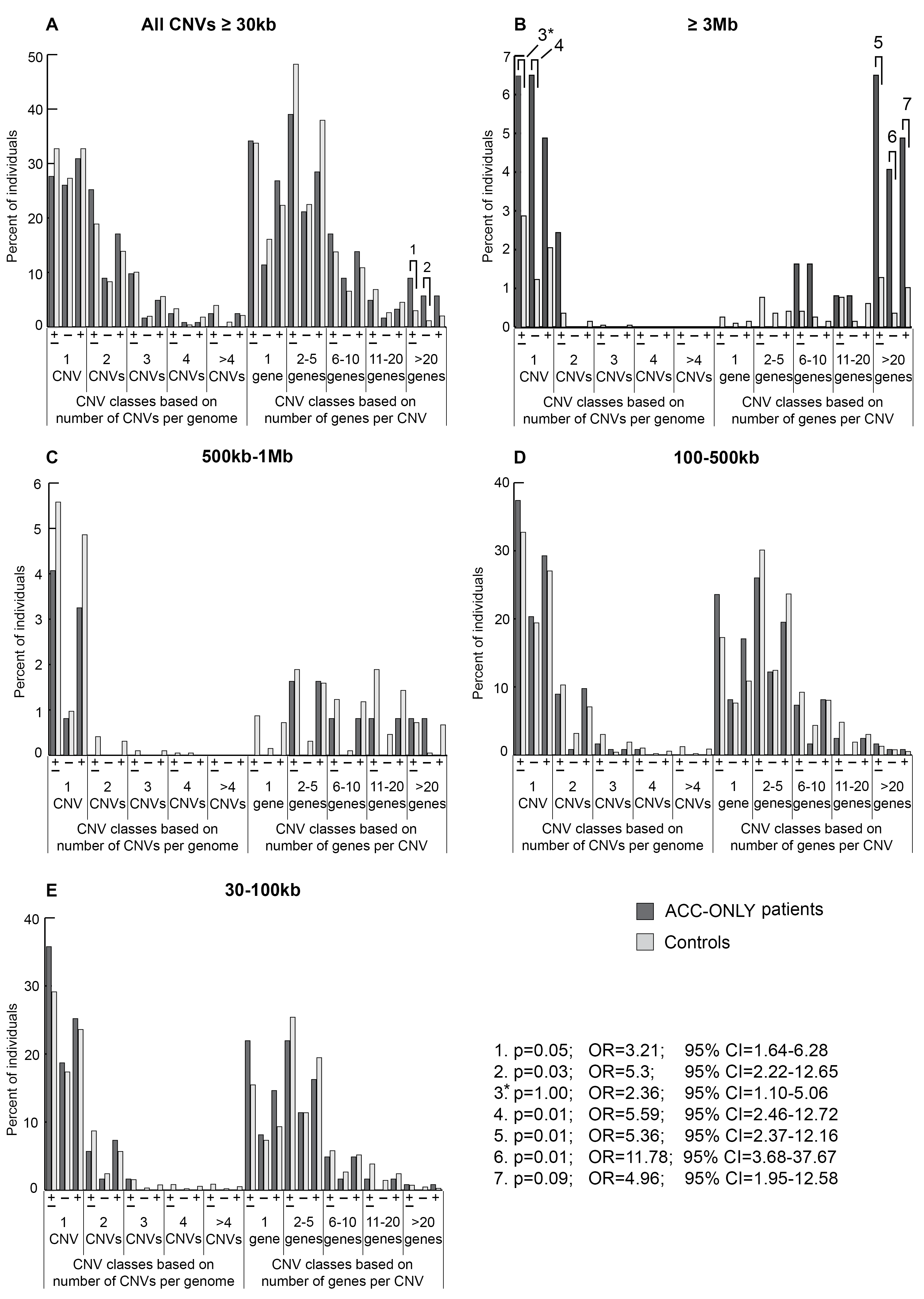

Supplement: Figure S4 — Genome-wide burden of rare CNVs of various sub-classes based on CNV size, number of exonic genes impacted, and number of CNVs per genome in 121 ACC-ONLY patients and 1,953 controls of Caucasian ethnicity. Deletions and duplications analyzed together are shown by “±”, deletions analyzed separately by “−”, and duplications analyzed separately by “+”. Panel A shows sub-categories based on number of genes and number of CNVs per genome for all rare CNVs≥30 kb regardless of size. Panels B–E show these same sub-categories for various size classes of rare CNVs. These size classes are: rare CNVs that were at least 1 Mb (≥1 Mb; panel B), those that were at least 500 kb but less than 1 Mb (500 kb–1 Mb; panel C), those that were at least 100 kb but less than 500 kb (100–500 kb; panel D), and those that were at least 30 kb but less than 100 kb (30–100 kb; panel E). Significant differences between patients (dark bars) and controls (light bars) are shown by black lines/hooks that connect patients and controls with numbers listed above. The numbers correspond to corrected p-values, odds ratios (OR), and 95% confidence intervals (CI) provided in the lower right. Asterisk: while the corrected p-value was not significant (1.00), the odds ratio (2.36) and 95% confident interval (1.10–5.06) were both highly suggestive of a significant difference. (TIF) [file pgen.1003823.s004.tif]

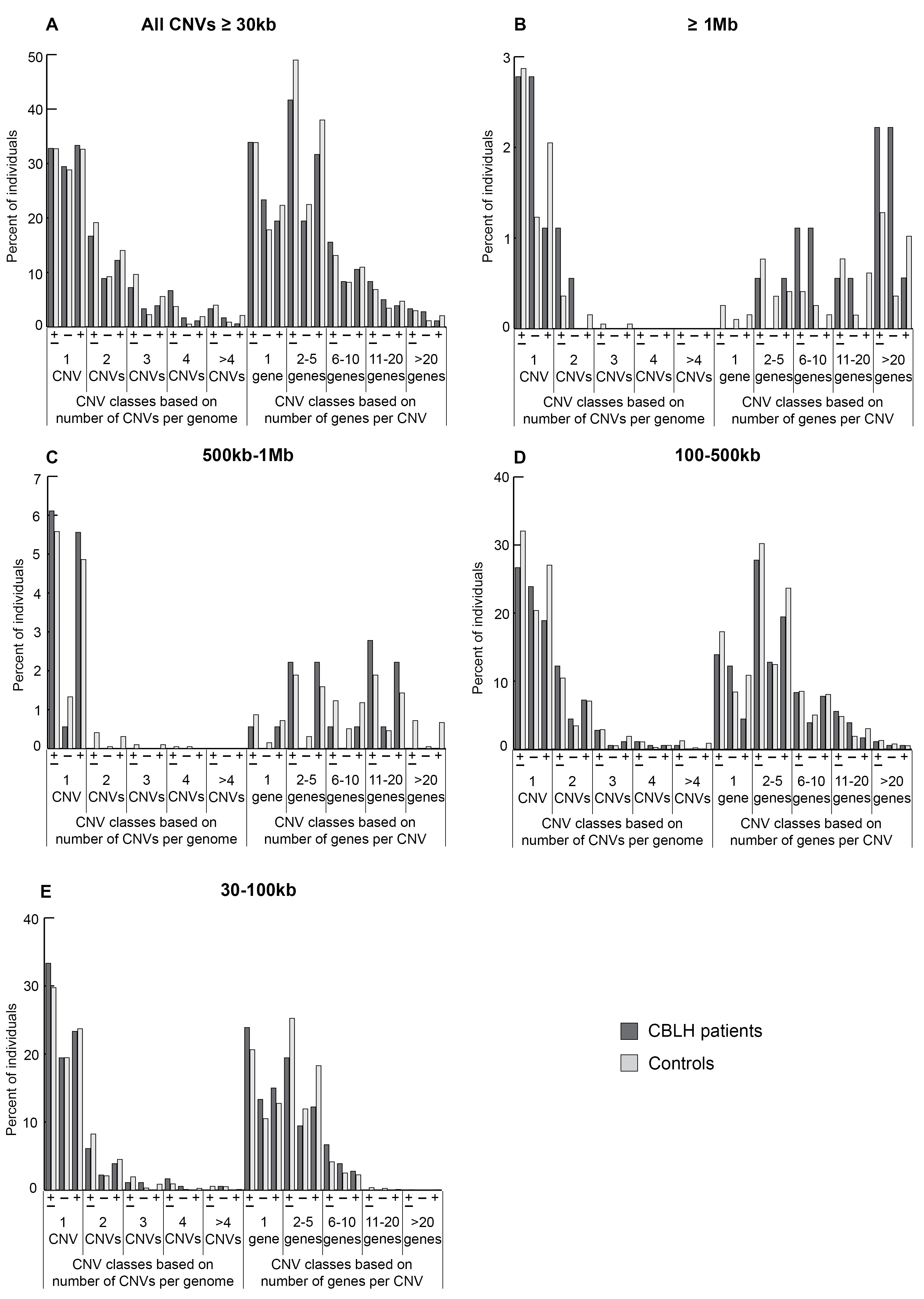

Supplement: Figure S5 — Genome-wide burden of rare CNVs of various sub-classes based on CNV size, number of exonic genes impacted, and number of CNVs per genome in 180 CBLH patients and 1,953 controls of Caucasian ethnicity. No significant differences were observed between patients and controls in any CNV category analyzed. Deletions and duplications analyzed together are shown by “±”, deletions analyzed separately by “−”, and duplications analyzed separately by “+”. Panel A shows sub-categories based on number of genes and number of CNVs per genome for all rare CNVs≥30 kb regardless of size. Panels B–E show these same sub-categories for various size classes of rare CNVs. These size classes are: rare CNVs that were at least 1 Mb (≥1 Mb; panel B), those that were at least 500 kb but less than 1 Mb (500 kb–1 Mb; panel C), those that were at least 100 kb but less than 500 kb (100–500 kb; panel D), and those that were at least 30 kb but less than 100 kb (30–100 kb; panel E). (TIF) [file pgen.1003823.s005.tif]

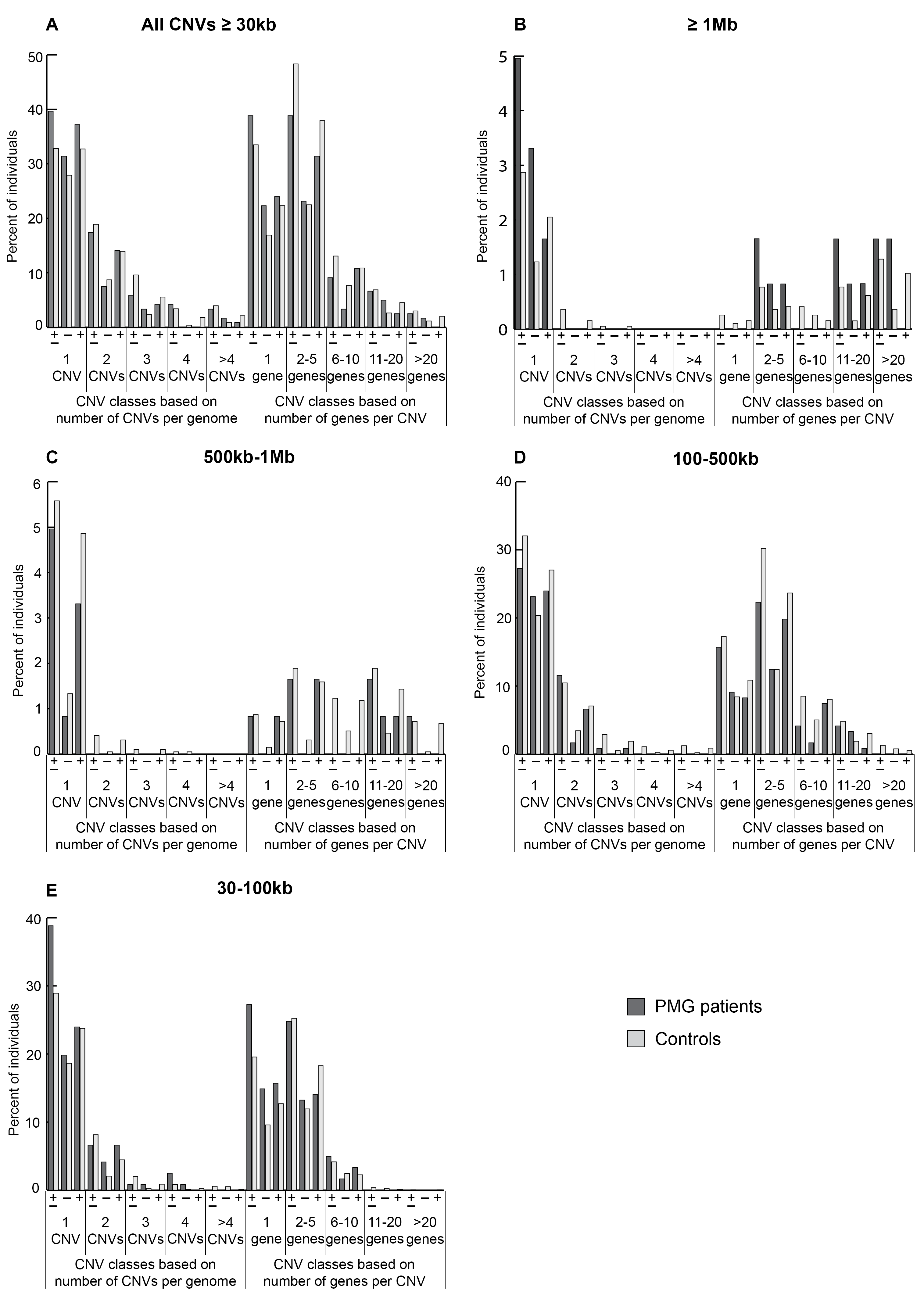

Supplement: Figure S6 — Genome-wide burden of rare CNVs of various sub-classes based on CNV size, number of exonic genes impacted, and number of CNVs per genome in 121 PMG patients and 1,953 controls of Caucasian ethnicity. No significant differences were observed between patients and controls in any CNV category analyzed. Deletions and duplications analyzed together are shown by “±”, deletions analyzed separately by “−”, and duplications analyzed separately by “+”. Panel A shows sub-categories based on number of genes and number of CNVs per genome for all rare CNVs≥30 kb regardless of size. Panels B–E show these same sub-categories for various size classes of rare CNVs. These size classes are: rare CNVs that were at least 1 Mb (≥1 Mb; panel B), those that were at least 500 kb but less than 1 Mb (500 kb–1 Mb; panel C), those that were at least 100 kb but less than 500 kb (100–500 kb; panel D), and those that were at least 30 kb but less than 100 kb (30–100 kb; panel E). (TIF) [file pgen.1003823.s006.tif]

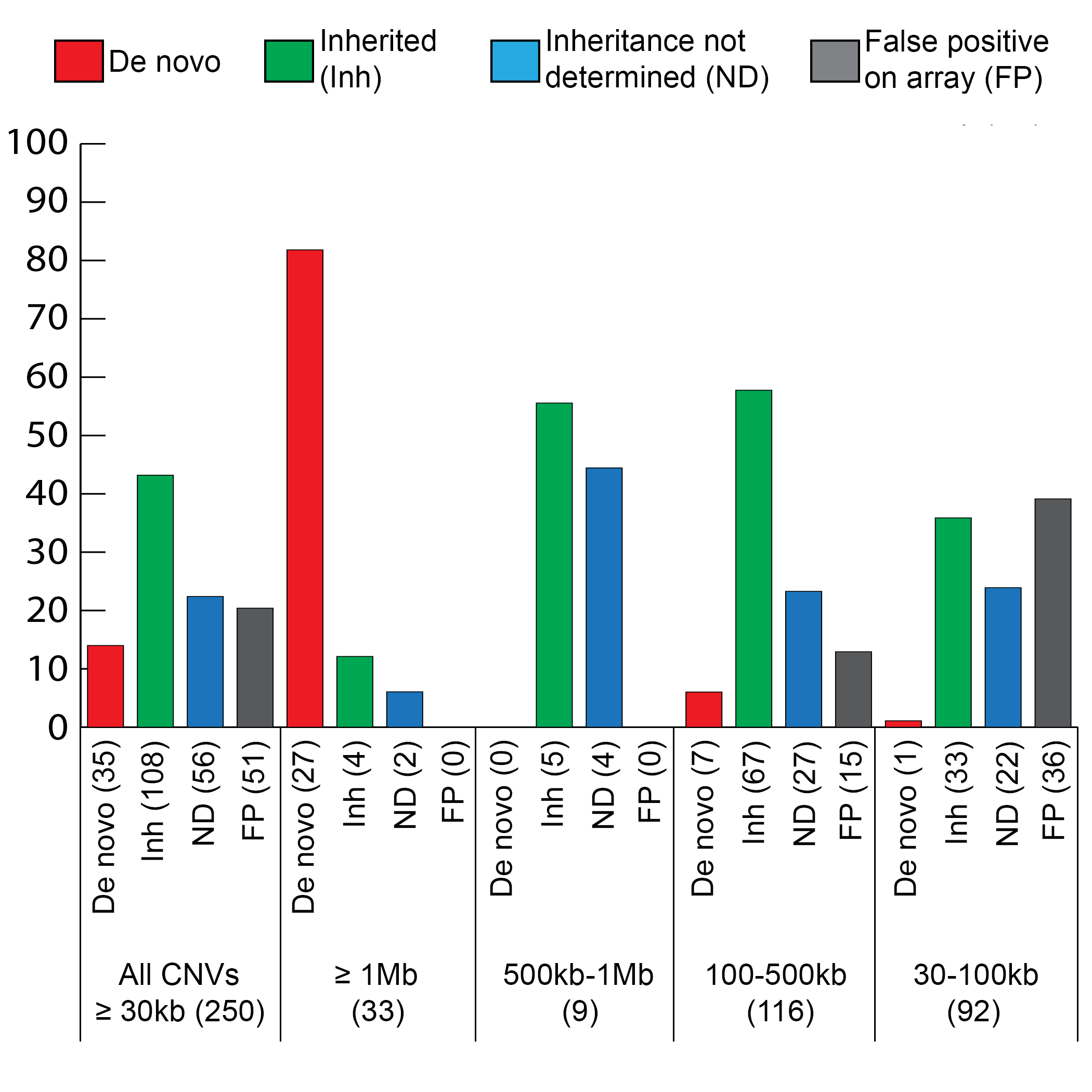

Supplement: Figure S7 — Summary of qPCR results showing the percent of all 250 patient-specific CNVs selected for independent confirmation. CNVs were classified as de novo, inherited (Inh), inheritance not determined (ND), and false positives on array (FP). These proportions are also provided for CNVs of different size classes, showing that smaller CNVs are more likely to be false positive array results. These size classes are: all patient-specific CNVs selected for qPCR confirmation that were at least 30 kb (≥30 kb), those that were at least 1 Mb (≥1 Mb), those that were at least 500 kb but less than 1 Mb (500 kb–1 Mb), those that were at least 100 kb but less than 500 kb (100–500 kb), and those that were at least 30 kb but less than 100 kb (30–100 kb). Table S5 provides a detailed listing of each CNV tested by qPCR. (TIF) [file pgen.1003823.s007.tif]

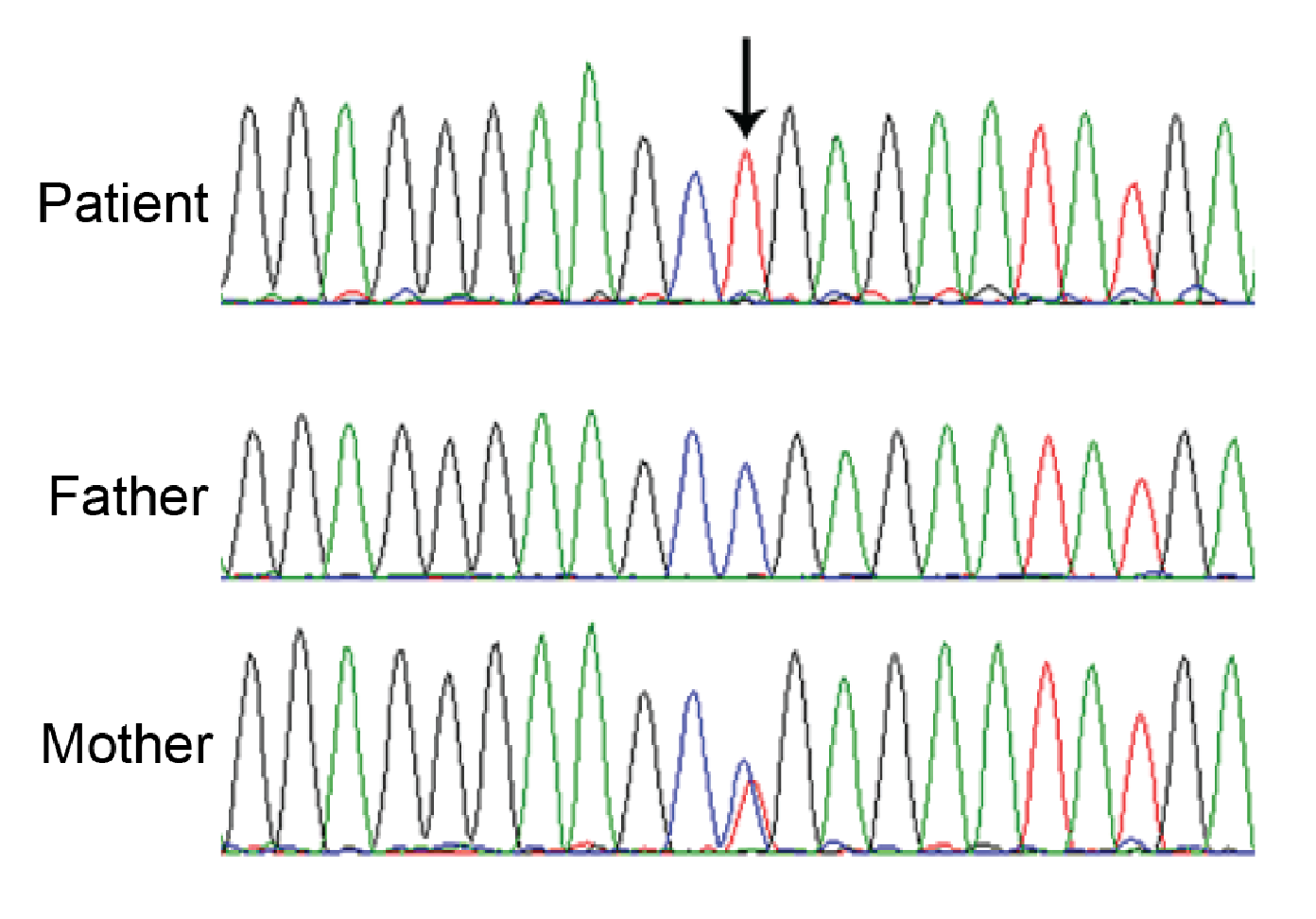

Supplement: Figure S8 — Sequence chromatogram of the non-deleted allele of NDE1 in a male patient (ID: LP97-141a1) diagnosed with ACC-PMG showing a maternally inherited nonsense p.R44X mutation at chr16:15761189 C/T (Hg19) shown by the arrow. (TIF) [file pgen.1003823.s008.tif]
